# Tumor necrosis factor receptor‐2 signaling pathways promote survival of cancer stem‐like CD133^+^ cells in clear cell renal carcinoma

**DOI:** 10.1096/fba.2019-00071

**Published:** 2020-01-03

**Authors:** John R. Bradley, Jun Wang, Simon Pacey, Anne Y. Warren, Jordan S. Pober, Rafia S. Al‐Lamki

**Affiliations:** ^1^ Department of Medicine NIHR Cambridge Biomedical Research Centre University of Cambridge Cambridge UK; ^2^ Department of Oncology NIHR Cambridge Biomedical Research Centre University of Cambridge Cambridge UK; ^3^ Department of Histopathology Addenbrooke's Hospital and University of Cambridge Cambridge UK; ^4^ Department of Immunobiology Yale University New Haven CT USA

**Keywords:** ccRCC, cell signaling, R1TNF, R2TNF, STAT3

## Abstract

Clear cell renal cell carcinoma (ccRCC) contains cancer stem‐like cells (CSCs) that express CD133 (ccRCC‐CD133^+^). CSCs are rarely in cell cycle and, as nonproliferating cells, resist most chemotherapeutic agents. Previously, we reported that tumor necrosis factor receptor‐2 (TNFR2) signaling promotes the cell cycle entry of ccRCC‐CD133^+^CSCs, rendering them susceptible to cell‐cycle‐dependent chemotherapeutics. Here, we describe a TNFR2‐activated signaling pathway in ccRCC‐^CD133+^CSCs that is required for cell survival. Wild‐type (wt)TNF or R2TNF but not R1TNF (TNF muteins that selectively bind to TNFR2 and TNFR1) induces phosphorylation of signal transducer and activator of transcription 3 (STAT3) on serine^727^ but not tyrosine^705^, resulting in pSTAT3^Ser727^ translocation to and colocalization with TNFR2 in mitochondria. R2TNF signaling activates a kinase cascade involving the phosphorylation of VEGFR2, PI‐3K, Akt, and mTORC. Inhibition of any of the kinases or siRNA knockdown of TNFR2 or STAT3 promotes cell death associated with mitochondrial morphological changes, cytochrome c release, generation of reactive oxygen species, and TUNEL^+^cells expressing phosphorylated mixed lineage kinase‐like (MLKL). Pretreatment with necrostatin‐1 is more protective than z‐VAD.fmk, suggesting that most death is necroptotic and TNFR2 signaling promotes cell survival by preventing mitochondrial‐mediated necroptosis. These data suggest that a TNFR2 selective agonist may offer a potential therapeutic strategy for ccRCC.

**Abbreviations**
Akt: protein kinase B
AZD5363: Akt protein kinase inhibitor
BMK120: buparlisib, PI‐3K inhibitor
CCK‐8: cell Counting Kit‐8
ccRCC: clear cell renal cell carcinoma
CLSM: confocal laser scanning microscopy
CSC: cancer stem‐like cells
ETC: electron transport chain
Etk: endothelial/epithelial nonreceptor tyrosine kinase
IF: immunofluorescence
JAK: Janus kinase
MFI: mean fluorescence intensity
MLKL: mixed lineage kinase‐like
mTOR: mammalian Target of Rapamycin
PE: phycoerythrin
PI‐3K: phosphatidylinositol 3‐kinases
pSTAT3^Ser727^: phosphorylated transducer and activator of transcription 3 at Serine727
pSTAT3^Ty705^: phosphorylated transducer and activator of transcription 3 at Tyrosine705
R1TNF: TNFR1 selective mutein
R2TNF: TNFR2 selective mutein
ROS: reactive oxygen species
SCID: severe‐combined immunodeficient mice
STAT3: signal transducer and activator of transcription 3
SU5408: VEGFR2 Kinase Inhibitor I
TBEA: typan bue exclusion assay
TNF: tumor necrosis factor‐α
TNFR1: tumor necrosis factor receptor 1
TNFR2: tumor necrosis factor receptor 2
TUNEL: terminal deoxynucleotidyl transferase (TdT) dUTP nick‐end labeling
VEGFR2: vascular endothelial growth factor receptor‐2
wtTNF: wild‐type tumor necrosis factor
z‐VAD.fmk: carbobenzoxy‐valyl‐alanyl‐aspartyl‐[O‐ methyl]‐ fluoromethylketone

## 1 INTRODUCTION

Tumor necrosis factor‐α (TNF) elicits a wide range of responses in almost all cell types, including cell death, survival, differentiation, and proliferation.^1^ TNF binds to either of two cell surface receptors; TNFR1 and TNFR2, which mediate distinct signaling pathways.^2^, ^3^, ^4^ The cell surface expression of each receptor is highly and independently regulated, and may determine the dominant response. TNFR1 can promote cell death,^2^ while TNFR2 can activate a pathway involving endothelial/epithelial nonreceptor tyrosine kinase (Etk), implicated in cell adhesion, migration, proliferation, and survival.^4^ TNFR2‐mediated activation of Etk in endothelial cells or renal cell carcinoma cells (RCC) results in tyrosine phosphorylation within the cytoplasmic sequences of vascular endothelial growth factor receptor‐2 (VEGFR2), resulting in a VEGF‐independent VEGFR2‐mediated response involving activation of a PI‐3K and Akt.^3^, ^5^ TNFR2 may also localize to mitochondria,^6^ but its function within this organelle is unknown.

Emerging evidence has shown that the capacity of some tumors to grow and propagate resides in a small population of slowly proliferating tumor cells referred to as cancer stem cells (CSCs).^7^ CSCs possess the characteristics of self‐renewal and are resistant to chemotherapy and radiotherapy. As they persist after cytotoxic treatment, they can give rise through cell division and differentiation to tumor cell progeny and tumor recurrence.^7^ Based on these CSCs properties, it has been proposed that their elimination can halt neoplastic expansion.^8^ CD133, alone or in combination with other molecular markers, has been used to isolate stem cells from normal kidney and CSCs from RCC*.* 9 We refer to the CD133^+^CSCs in our study as “cancer stem‐like cells” because although previous studies have reported that CD133^+^cells from several human tumors exhibit xenotransplantation potential in severe combined immunodeficiency (SCID) mice,^10^, ^11^ isolated CD133^+^cells from human RCC tissue failed to form tumors when transplanted into SCID mice but instead, these cells potentiate tumor engraftment when cotransplanted with CD133^‐^RCC cells.^11^ Based on these data, we are uncertain if they are truly cancer stem cells capable of differentiation into more mature RCC cells even though they express stem cell markers. Significantly, CD133 expression has been suggested as a useful risk stratification tool in RCC.^12^

Signal transducers and activators of transcription (STAT) exist as latent, un‐phosphorylated monomers in the cytoplasm, and transmit signals from diverse cytokines and growth factors.^13^ STAT proteins can be tyrosine, serine, and/or threonine phosphorylated.^14^ The best‐known pathway involves the Janus kinase (JAK)‐mediated phosphorylation of tyrosine residues, inducing STAT dimerization and translocation into the nucleus where phosphorylated (p)STATs act as transcription factors.^15^ For STAT3, JAK signaling is typically activated in response to cytokines that bind to receptors containing gp130, inducing the JAK‐mediated phosphorylation of tyrosine‐705.^15^ Nuclear phosphorylation of STAT3 tyrosine‐Y705 (pSTAT3^Ty705^) promotes cell growth and many malignancies exhibit constitutive pSTAT3^Ty705^. Neither TNF receptor has been linked to this response. Recent evidence has shown that phosphorylation of STAT3 on serine‐727 (pSTAT3^Ser727^) also contributes to, or is sufficient for, the growth and transformation of malignant cells.^16^, ^17^ pSTAT3^Ser727^ has been identified in mitochondria,^16^, ^17^, ^18^ and pSTAT3^Ser727^ but not pSTAT3^Ty705^ is crucial for the optimal activity of complexes of the electron transport chain (ETC) in mitochondria where it protects cells against the stress‐induced generation of reactive oxygen species (ROS).^19^, ^20^

We recently reported that selective ligation of TNFR2 can drive the cell‐cycle entry of CD133^+^CSCs isolated from ccRCC (ccRCC‐^CD133+^CSCs), rendering them sensitive to killing by a chemotherapeutic agent.^21^ The current investigation was initiated to understand the mechanism through which TNFR2 acts. We describe an unexpected link between TNFR2 and STAT3 signaling in ccRCC‐CSCs that express CD133 and other stem cell markers. We report that in CD133^+^CSCs, TNFR2 signaling through the VEGFR2/Phosphoinositide 3‐kinase (PI‐3K)/Protein kinase‐B (Akt)/Mechanistic Target of Rapamycin complex (mTORC) pathway induces pSTAT3^Ser727^ that colocalizes with TNFR2 in mitochondria. Disruption of this pathway results in mitochondrial instability, mitochondrial outer membrane permeabilization, generation of ROS, and necroptotic cell death.

## 2 MATERIALS AND METHODS

### 2.1 Reagents/Antibodies

Antibodies and reagents used were anti‐CD133/1‐W6B3C1 (cat~130‐092‐395) (Miltenyi Biotec Ltd, Surrey, UK), anti‐CD133 (cat~E90032)(Source Bioscience, Nottingham, UK), anti‐STAT3 (phospho‐^Ty705^) [EP2147Y] (ab76315), anti‐STAT3 (phospho‐^Ser727^) (ab131103), anti‐PI‐3K‐^p110β^ [epr5515(2)](cat~ab151549), anti‐Akt (phospho‐^Thr308^) (cat~ab8933), anti‐Akt (phospho‐^Ser473^)[EP2109Y](cat~ab81283), anti‐mTOR (phospho‐^Ser2448^)(cat~ab118815), anti‐VEGFR2 (phospho‐^Y1054‐1059^)(cat~ab5473), anti‐human TNF Receptor II [EPR1653] (cat~ab109322), anti‐phospho‐Histone H3^S10^ antibodies (cat~ab14955 and cat~ab5176), anti‐phospho‐MLKL^Ser358^ (cat~ab187091) and N‐Acetyl‐L‐Cysteine (NAC, cat~ab143032), and phosphorylated JAK‐1, −2, and −3 (cat~ab32101, ab61102; ab138005; Abcam). Rabbit anti‐human mitochondria (MAB3598) (Millipore Ltd). Anti‐STAT3 (cat~9132), Anti‐phospho‐STAT3 ^Ser727^ [M9C6] (cat~4113), anti‐VDAC [D73D12] (cat~4661) and anti‐cleaved caspase‐3^Asp175^ (cat~9661), and phycoerythrin (PE)‐conjugated anti‐cleaved caspase‐3^p175^ [5A1E] (cat~9978) (Cell Signaling Technology). Anti‐TNF RII/TNFRSF1B (cat~MAB226‐PB) (R&D Systems). Anti‐SSEA‐4 [MC81370] (cat~MA1021X) (Perbio Science Ltd). Anti‐Nanog (cat~MCA5657T) (Bio‐Rad Laboratories Ltd). Anti‐cytochrome c (cat~556432), PE‐conjugated: anti‐phospho‐STAT3^S727^ (cat~558557), anti‐mTOR^Ser2448^ (cat~563489) and anti‐phospho‐Akt^Threonine308^ (cat~558275)(BD Pharmingen). MitoTracker™Red CMXRos (cat~M7512), Hoechst‐33342 (cat~62249), 8‐well Nunc™ Lab‐Tek™ II Chamber Slides™ System (cat~154526PK), Gibco™ Opti‐MEM™ I Reduced Serum Medium (cat~31985062), CellROX™Green (cat~C10492), BD Cytofix™ (cat~554655), and BD Phosflow Perm Buffer II (cat~558050) (Thermofisher Scientific). Tissue Dissociation Kit (cat~130‐095‐929), MS columns (cat~130‐042‐201), and C‐Tubes (cat~130‐093‐237) (Miltenyi Biotec). z‐VAD.fmk (cat~G7231; Promega Madison). VEGFR2 Kinase Inhibitor I (SU5408, cat~ab145888), Ku‐0063794 (mTORC1/2 inhibitor) (cat~13597) (Cayman Chemical), Akt inhibitor (AZD5363) (cat~S8019), and PI‐3K inhibitor (BMK120, Buparlisib) (cat~S2247)( Selleckchem). wtTNF (cat~201‐TA‐020; R&D Systems), TNFR1 and TNFR2‐muteins (R1TNF and R2TNF) 3, 4, 21 are a generous gift from Prof Peter Vandenabeele (Ghent, Belgium). For TNFR1 the point mutation is R32W and for TNFR2 is D143N.^22^ Necrostatin‐1 (Nec‐1, cat~N9037) (Sigma‐Aldrich, Dorset, UK), TUNEL‐label (dUTP^−FITC^) (cat~11767291910), Terminal transferase enzyme (TdT) (cat~03333566001), Cell Counting Kit‐8 (cat~96992), and trypan blue dye (cat~T6146) (Sigma‐Aldrich, Dorset, UK).

### 2.2 Collection of tissue samples

Samples of ccRCC and adjacent nontumor kidney tissue (NK) (n* *= 10) were collected from radical nephrectomies removed for tumor resection through Cambridge University Hospital Tissue Bank. Written informed consent was obtained from all patients in accordance with the local Ethics Committee. ccRCC samples were graded on hematoxylin and eosin (H&E) stained sections by AYW according to the World Health Organisation/International Society of Urological Pathology (ISUP).^23^ All samples were immersed in tissue culture medium as previously described 2 and processed for the isolation of stem cells from ccRCC (ccRCC^CD133+^ CSCs) and NK (NK‐^CD133+^cells) and short‐term organ culture experiments.^2^

### 2.3 ccRCC and NK organ cultures

As previously described,^4^ duplicate <1 mm^3^ fragments of ccRCC (grade 2) and NK were maintained in organ culture in 96‐well plates and either left in medium alone (UT) or pretreated with wild‐type (wt)TNF (10 ng/mL) or R1TNF or R2TNF (1 μg/mL) for 3h at 37°C. As previously reported,^4^ TNF does not induce any significant effect in organ cultures at <3 hours with a longer incubation time resulting in rapid deterioration of tissue quality. About 5 μmol/L thick formaldehyde fixed paraffin‐wax embedded sections was then prepared for subsequent experiments.

### 2.4 Isolation and characterization of ccRCC‐^CD133+^ CSCs and NK‐^CD133+^ cells

CD133^+^cells from ccRCC and NK tissue were isolated as previously described.^21^ In brief, ~2 cm^2^ tissue pieces were digested into single‐cell suspension using Miltenyi Tissue Dissociation Kit on a GentleMACs Disassociator and incubated for 30min (37°C) on a MACsMix rotator, then passed through a 40 μm strainer. Cells were cultured in nondifferentiating medium.^11^ ccRCC‐^CD133+^CSCs and NK^CD133+^ cells were isolated using immunobeads conjugated to CD133 antibody and passed through the LS column on MiniMACS Separator.^24^ Both magnetically labeled cells (CD133^+^cells) and unlabelled cells (CD133‐cells) were collected. A yield of ~1 × 10^4^ cells/tissue sample was seeded in T25 flasks and grown to confluence for subsequent experiments.

### 2.5 RNA interference (siRNA)

TNFR2 and STAT3 expression were knocked down in ccRCC^CD133+^ CSCs and NK^CD133+^ cells using small interfering RNA (siRNA) (Horizon Discovery Ltd). These included human ON‐TARGETplus™ TNFRSF1B siRNAs (10 nMol‐set of four individual, cat~J‐003934‐05‐0002, J‐003934‐06‐0002, J‐003934‐07‐0002, J‐003934‐08‐0002), human ON‐TARGETplus™TNFRSF1A (10 nMol‐set of four individual, cat~L‐005197‐00‐0005), and ON‐TARGETplus™ nontargeting human siRNA (NTsiRNA; cat~D‐001810‐01‐05), which does not target any known mammalian genes, as negative control, set of three human individual Stealth STAT3siRNAs (20nMol, cat~HSS110279, HSS186130, HSS186131) (Invitrogen). siRNA complexes (50 nmol/L) were transfected using DharmaFECT Duo (5 μmol/L, cat~T‐2010‐03)(Horizon Discovery) in Opti‐MEM™ Reduced Serum Medium (Thermofisher Scientific), respectively, for 24, 48, and 72h (37°C) and knockdown efficiency determined by immunoblotting.

### 2.6 Immunofluorescence (IF) and confocal laser scanning microscopy (CLSM)

Immunostaining was carried out as previously described.^21^ For organ cultures, deparaffinized sections were exposed to high‐pressure antigen retrieval before incubation with anti‐pSTAT3^Ser727^, ‐CD133 or ‐TNFR2 (1:100) overnight (4°C). For cells, 8‐well slide chambers were permeabilized at −20°C in cold methanol (4 minutes) before incubation with anti‐CD133 alone or ‐anti‐SSAE and ‐Nanog (1:100 dilution) overnight (4°C). In parallel, cells treated with wtTNF (10 ng/mL), R1TNF, R2TNF (1 μg/mL) or left UT for 30 minutes (37°C) were incubation with anti‐pSTAT3^Ser727^ and ‐CD133 or ‐TNFR2; anti‐CD133 and phosphorylated‐VEGFR2^Y1059^, ‐PI‐3K^110β^, ‐Akt^Thr308^ or ‐mTOR^Ser2448^. Following TNF treatment, some cultures were incubated with MitoTracker™Red CMXRos (5 μmol/L)(detects mitochondria in live cells) for 15 minutes (37°C) then fixed in cold methanol prior to immunostaining for TNFR2 or pSTAT3^Ser727^. For siRNA transfected cells, MitoTracker™Red CMXRos was followed by incubation with anti‐Cytochrome‐c antibody. This was followed by specified‐specific secondary antibody‐conjugated to Northern Light‐^498^ or −^557^ plus Hoechst 333 342 (1 μg/mL) for nuclei detection for 1 hour (1:100), mounted in VectaShield (Vector Laboratories) before viewing on a TCS‐SPE CLSM (Leica Microsystems). Isotype‐specific sera was used as a negative control. Image for each fluorophore was acquired sequentially using the same constant acquisition time and settings rather than simultaneously to avoid crosstalk between channels. Images were processed using Adobe Photoshop CS6 software and the Mean Fluorescence Intensity (MFI) minus the background signal was quantified using Image J 1.49v. For TNFR2/pSTAT3^Ser727^, double‐positive cells were counted in random 10 high power fields of view in an unbiased manner and presented as the percentage using the formulae (double‐positive cells/total cells ×100).

### 2.7 Flow cytometry

About 1x10^5^cells/well were treated with wtTNF R1TNF, R2TNF or left UT for 30 minutes (37°C), fixed in BD Cytofix™ (room temperature) and permeabilized in BD Phosflow Perm Buffer II (4°C) for 10 minutes in each, then incubated with PE‐conjugated antibody to ‐CD133, ‐TNFR2, ‐pSTAT3^Ser727^, ^‐^VEGFR2^Y1059^, ‐PI‐3K^110β^, ‐Akt^Thr308^ or ‐mTOR^Ser2448^ for 15 minutes (room temperature) and examined on BD FACSCanto™II. Parallel cultures were pretreated for 1h (37°C) with specific inhibitors to these phosphorylated proteins: [SU5408 (VEGFR2)(1 μmol/L), BMK120 (PI‐3K)(4 μmol/L), AZD5363 (Akt) (0.8 μmol/L), and Ku‐0063794 (mTORC1/2] (5 μmol/L)^25^, ^26^, ^27^, ^28^, ^29^ prior to treatment with wtTNF, R1TNF or R2TNF or left in vehicle (dimethyl sulfoxide, DMSO; <0.2%) referred, herein, as UT. The inhibitors were diluted in DMSO to a stock concentration of 10mm and stored at −20°C. The working concentration for each inhibitor was determined using a dose‐response curve and controls included treatment of cells with inhibitors alone to determine any nontarget effects. A total of 20 000 events per sample were acquired and the data were analyzed with FlowJo v10 software.

### 2.8 Immunoblotting

About 0.5x10^6^ cells/well were treated with wtTNF or UT for 30 minutes (37°C) than lysed in RIPA buffer (Sigma Aldrich). About 50 μg protein separated by SDS‐PAGE was blotted on nitrocellulose membrane and incubated with anti‐total STAT3 or ‐pSTAT3^Ser727^ overnight (4°C) (1:1000). Blots were incubated with species‐specific HRP‐conjugated secondary antibodies for 1h (room temperature) (Dakocytomation) and protein bands detected by Super Signal West Pico Chemiluminescent substrate kit (Thermo Fisher Scientific) and visualized using ChemDoc Universal Hood III Imaging System (Bio‐Rad Laboratories Ltd). β‐Actin was used as a loading control. Cells were processed in a similar manner following siRNA transfection.

### 2.9 Cell viability and cell death assays

Cell counting kit‐8 (CCK‐8) and Trypan Blue Dye Exclusion (TBDE) assays were used to analyze cell viability following siRNA transfection targeting TNFR2, STAT3, NTsiRNA plus DharmaFECT‐Duo or left UT in Opti‐MEM™ Reduced Serum Medium for 24, 48, and 72 hours (37°C). Following siRNA transfection, some cells were posttreated with wtTNF, R1TNF, and R2TNF for 30 minutes (37°C) then CCK‐8 reagent (10 μL) was added to each well for 4h (37°C) and the absorbance (OD) was measured at 450nm on Infinite M200 PRO Microplate Reader (Tecan). Cell viability was calculated using the formula (OD of test samples/OD of control ×100). Parallel cultures were stained with 0.2% Trypan Blue Dye, viable/nonviable cells counted using a hemocytometer, and percentage cytotoxicity calculated using the formula (Live or Dead cell count/Total cells ×100).

TUNEL (Terminal deoxynucleotidyl transferase (TdT) dUTP nick‐end labeling) was used to detect cell death as previously described.^2^, ^4^ Following siRNA transfection for 24, 48, and 72 hours, cells were treated with wtTNF, R1TNF, R2TNF or left UT for 30 minutes (37°C) then fixed in cold methanol for 4 minutes (−20°C) and incubated in a mixture of TUNEL^FITC^‐label and TdT‐enzyme (Sigma‐Aldrich) for 45 minutes (37°C). This was followed by Hoechst 33342 for 10 minutes (room temperature) and mounting in Vectashield before viewing on CLSM. The number of TUNEL^+^cells per total number of unstained viable cells were counted in random 10 high power fields of view (x40 magnification) in an unbiased manner to determine the percentage of TUNEL^+^cells. To evaluate apoptotic and necroptotic cell death, following TUNEL, cells were immunostained with antibody to cleaved caspase‐3^Asp175^ (a marker for apoptosis) or phosphorylated‐MLKL (pMLKL^Ser358^; a marker for necroptosis) (1:100) (4°C) followed by species‐specific secondary antibody (1:200) for 1 hour (room temperature). Parallel cultures were pretreated with z‐VAD.fmk (blocks apoptosis) (20 μmol/L) or Nec‐1 (30 μmol/L) (blocks RIPK1, upstream of MLKL)^21^ either alone or in combination for 1h prior to siRNA transfection then posttreated with or without wtTNF or left UT for 30 minutes (37°C) prior to TUNEL and IF using the same antibodies.

### 2.10 *Transmission electron microscopy (TEM*)

As we have previously described,^30^ after siRNA targeting TNFR2 or STAT3 or NTsiRNA or left UT for 72 hours (37°C), 0.5 × 10^6^ cells/well seeded on 6‐well plates were fixed in 2% glutaraldehyde/2% formaldehyde, osmicated in 1% Osmium tetroxide, dehydration in ascending series of ethanol solutions, and embedded in Quetol 651 resin before viewing in a Hitachi Capital (UK) PLC, Leeds West Yorkshire at an accelerating voltage of 80 kV.

### 2.11 Mitochondria Isolation

ccRCC‐^CD133+^CSCs (1.5 × 10^6^cells) were treated with R2TNF (1 μg/mL) or left UT for 30 minutes (37°C) then mitochondrial fraction was isolated using Mitochondria Isolation Kit (cat~89874; Thermofisher Scientific). As we have previously described,^31^ for immunogold electron microscopy (IGEM), cells were cells stained for TNFR2 and pSTAT3^Ser727^ (1:5) overnight and further stained with 20 and 5nm gold particles (British Biocell, Cardiff, UK) (1:100), followed by 2% phosphotungstic acid pH 7.0 (30 seconds) before viewing on TEM. As we were unable to obtain sufficient mitochondria fraction for immunoblotting from ccRCC‐^CD133+^CSCs, we used a human RCC immortalized cell line (RCC‐26; derived from a patient with stage I disease) 32 Equal amount of protein (50µg) from mitochondria and cytosolic fraction were probed with anti‐ TNFR2, ‐total STAT3 or ‐pSTAT3^Ser727^ followed by specific HRP‐conjugated secondary antibodies prior to visualization.

### 2.12 Measurement of intracellular ROS

Following siRNA transfection for 24, 48, and 72 hours (37°C), 5 × 10^5^ cells/well were processed for the measurement of intracellular ROS using the cell‐permeable CellROX™ Green^33^, ^34^ (Thermo Fisher Scientific), a fluorogenic probe that exhibits bright‐green photostable fluorescence upon oxidation. Negative control included pretreatment with N‐acetylcysteine (NAC‐ 5 mmol/L, ROS scavenger) for 2 hours (37°C) prior to siRNA transfection and positive control involved treatment with ROS inducer; tert‐butyl hydroperoxide (TBHP‐5 mmol/L) for 1 hour (37°C) prior to staining. CellROX® Green reagent (500 nmol/L) was added to each well for 30 minutes (37°C) and immediately analyzed by flow cytometry. The fluorescent signal proportional to cellular ROS levels was excited by laser at 488nm and quantified on a BD FACS Canto II. In parallel, cells were grown on 8‐well chamber slides, treated, and stained in a similar manner before viewing on CLSM. Images were transferred to Image J for quantification and the percentage of CellROX®Green positive cells was calculated as [positive cells/ total cells (×100)] in 10 random fields of view at ×40 magnification.

### 2.13 Statistical analysis

Data are expressed as mean ± SEM To account for patient‐specific differences, all experiments were performed with organ cultures and corresponding isolated cells from at least three patients with triplicate samples in each assay unless otherwise stated. Differences between two groups were analyzed by Student's *t* test and between >2 groups by one or two‐way analysis of variance followed by Bonferroni's post hoc test using GraphPad Prism v7.0 (San Diego). A *P* value <.05 was considered statistically significant.

## 3 RESULTS

### 3.1 TNFR2 ligation induces pSTAT3^Ser727^ but not pSTAT3^Ty705^ in CD133^+^cells of ccRCC in situ in organ culture and in isolated cells

**Figure 1:**
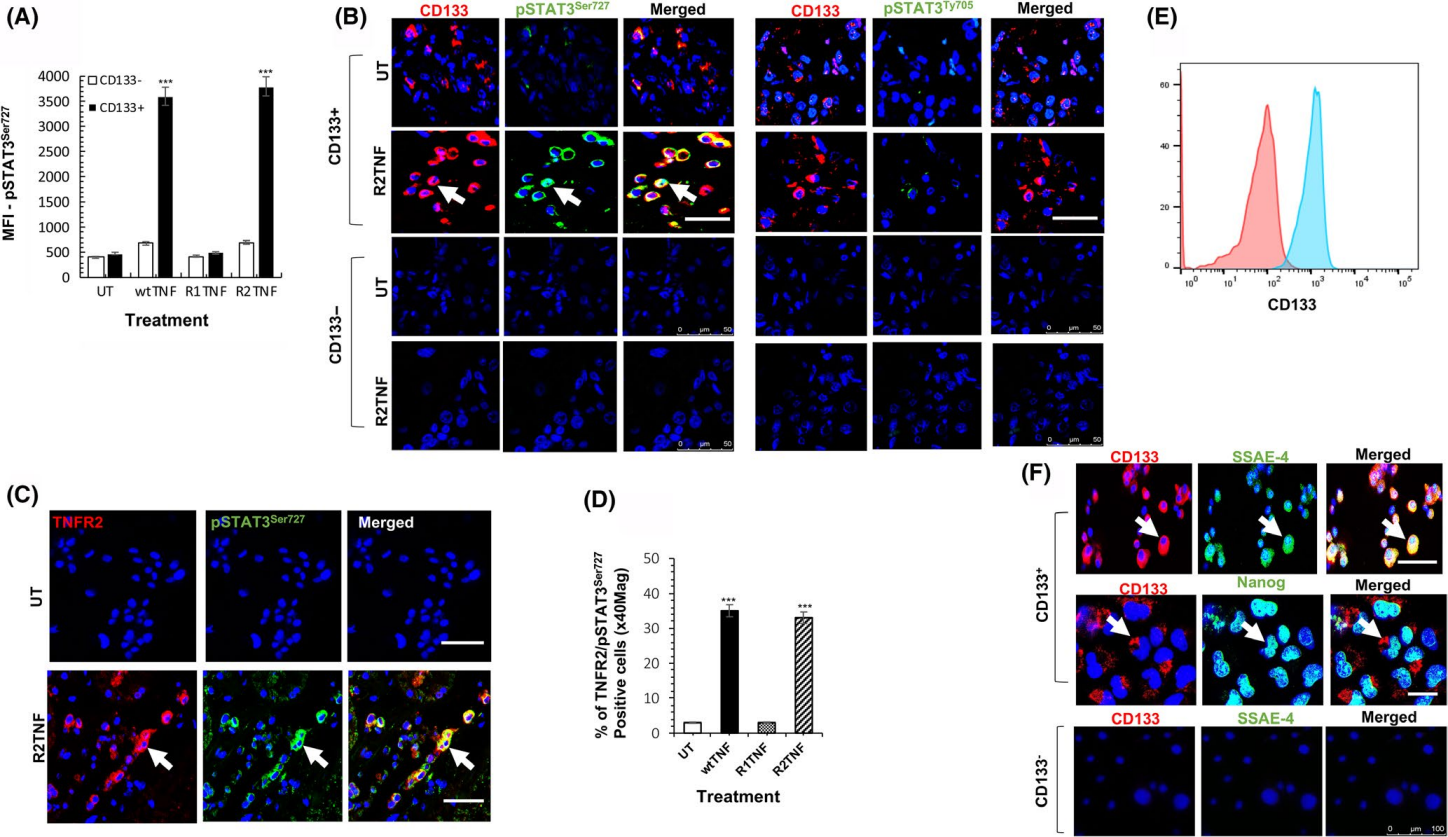
A‐D, Organ cultures ccRCC (grade 2) were treated with either wild type‐(wt)TNF, R1TNF or R2TNF or left untreated (UT‐in media alone) for 3h at 37°C then immunostained for STAT3 serine phosphorylation (pSTAT3^Ser727^) or tyrosine phosphorylation (pSTAT3^Ty705^) and CD133 or with TNFR2 and pSTAT3^Ser727^. A, Immunofluorescence data represented as median fluorescence intensity (MFI) shows wtTNF and R2TNF (not R1TNF) induction of pSTAT3^Ser727^ expression in CD133^+^ CSCs (but not CD133^‐^cells) as compared to UT control. B, Representative confocal images show of pSTAT3^Ser727^ but not pSTAT3^Ty705^ expression in resident CD133^+^CSCs (*arrows*), with some cells also positive for TNFR2 (*arrows*) in organ cultures treated with R2TNF. In contrast, no staining is detected in CD133^‐^cells. C, Quantified in (D) as the percentage of positive cells/total cells (×100) at ×40 Mag. (Error bars represent mean ± SEM; N = 5 independent experiments of different organ cultures per treatment group; ^***^ *P* < .001 vs UT; One way ANOVA). E, Flow cytometry analysis shows the CD133 expression in isolates of ccRCC‐^CD133+^ CSCs (F). CD133^+^CSCs (but not CD133^‐^cells) are positive for stem cell markers (SSEA‐4 or Nanog). Blue nuclei stained with Hoechst 33342. (N = 3 independent experiments of three different isolates with similar results). Mag ×63; Scale bars: 100 μmol/L

pSTAT3^Ty705^ associated with nuclear translocation is seen in many stem cells and malignancies and may play a role in cell proliferation. Although not known to be affected by TNF, we investigated if TNFR2 signaling, which is mitogenic in ccRCC, might activate this pathway in resident ^CD133+^CSCs in ccRCC organ cultures. R2TNF did not increase pSTAT3^Ty705^ but unexpectedly increased the expression of pSTAT3^Ser727^ by ~10‐fold as compared to UT controls, quantified as mean fluorescence intensity (Figure 1A) and representative confocal images as shown in Figure 1B. wtTNF (not R1TNF) showed similar findings. wtTNF or R2TNF (not R1TNF) also induced TNFR2 expression, which colocalized with pSTAT3^Ser727^ in ~ 35% of the cells (Figure 1C,D). To further confirm the absence of pSTAT3^Ty705^ expression after TNF‐treatment, organ cultures were immunostained for phosphorylated JAK‐1, ‐2, and ‐3. No signal for phosphorylated JAKs was detected in all cultures (**data not shown**).

**Figure 2:**
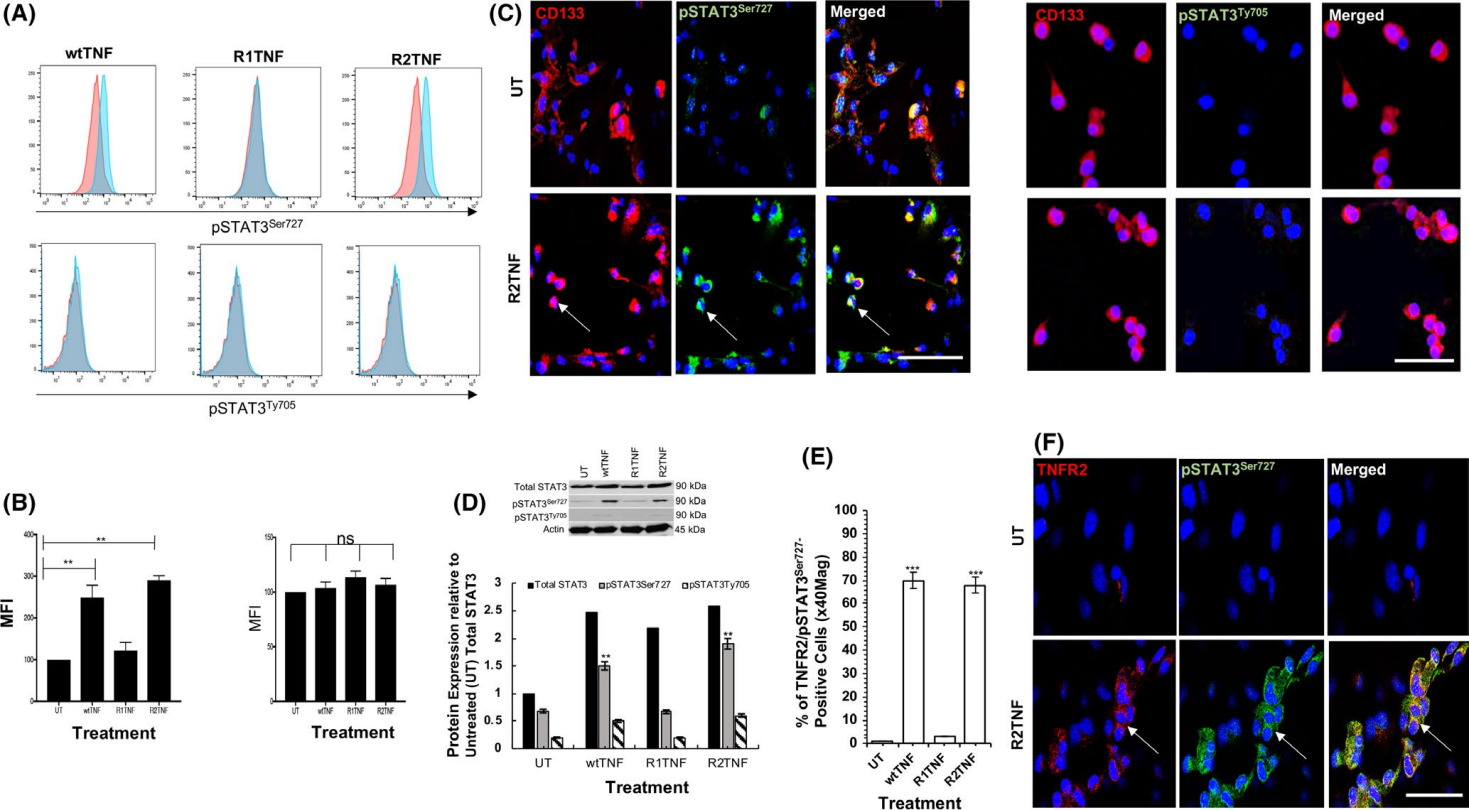
Isolates of ccRCC‐^CD133+^CSCs were treated with either wild type‐(wt)TNF, R1TNF or R2TNF or left untreated (UT‐in media alone) for 30 min at 37°C then immunostained for phosphorylated STAT3 serine (pSTAT3^Ser727^) or tyrosine (pSTAT3^Ty705^) and CD133 or TNFR2. A, Flow cytometry analysis quantified as median fluorescence intensity (MFI) show wtTNF and R2TNF (not R1TNF) induction of pSTAT3^Ser727^ but not pSTAT3^Ty705^ as compared to UT controls (B), confirmed by confocal microscopy, (C) and a representative immunoblot of whole‐cell lysates, relative to total STAT3 in UT cultures shown in (D). wtTNF and R2TNF also induce colocalization of TNFR2 and STAT3^Ser727^, quantified as percentage of positive cells/total number of cells (×100) at ×40 Mag (E) and, positive cells (*arrows*) illustrated by confocal microscopy showing cytoplasmic/granular pattern of staining (F). Blue nuclei stained with Hoechst 33342. Error bars represent mean ± SEM; N = 3 independent experiments of different isolates per treatment group. ^**^ *P* < .01 and ^***^ *P* < .0001 vs UT; ns‐not significant. One way ANOVA. Mag ×63, Scale bars: 100 μmol/L

To further study the effect of TNF, we isolated CD133^+^cells from ccRCC‐(ccRCC^CD133+^CSCs).^21^ Isolated cells were strongly positive for CD133 and exhibited a stem‐cell phenotype evidenced by strong immunostaining for SSEA‐4 and Nanog (Figure 1E,F), as previously reported.^21^ To test whether TNF phosphorylates STAT3 in ccRCC‐^CD133+^CSCs, cells were treated with wtTNF, R1TNF, and R2TNF or left UT for 30 minutes (37°C). Similar to ccRCC organ cultures, pSTAT3^Ser727^ but not pSTAT3^Ty705^ was dramatically increased by wtTNF or R2TNF (not R1TNF) detected by flow cytometry (Figure 2A, quantified in 2B), confirmed by IF‐confocal microscopy (Figure 2C), and immunoblot of whole‐cell lysates (Figure 2D). As in ccRCC organ cultures, R2TNF‐induced TNFR2 expression, which colocalized with pSTAT3^Ser727^ in ~68% of the cells showing a cytoplasmic/granular pattern of staining (Figure 2E,F).

### 3.2 TNFR2‐induced pSTAT3^Ser727^ is dependent upon a VEGFR2/PI‐3K/Akt/mTORC kinase cascade in ccRCC‐^CD133+^CSCs

**Figure 3:**
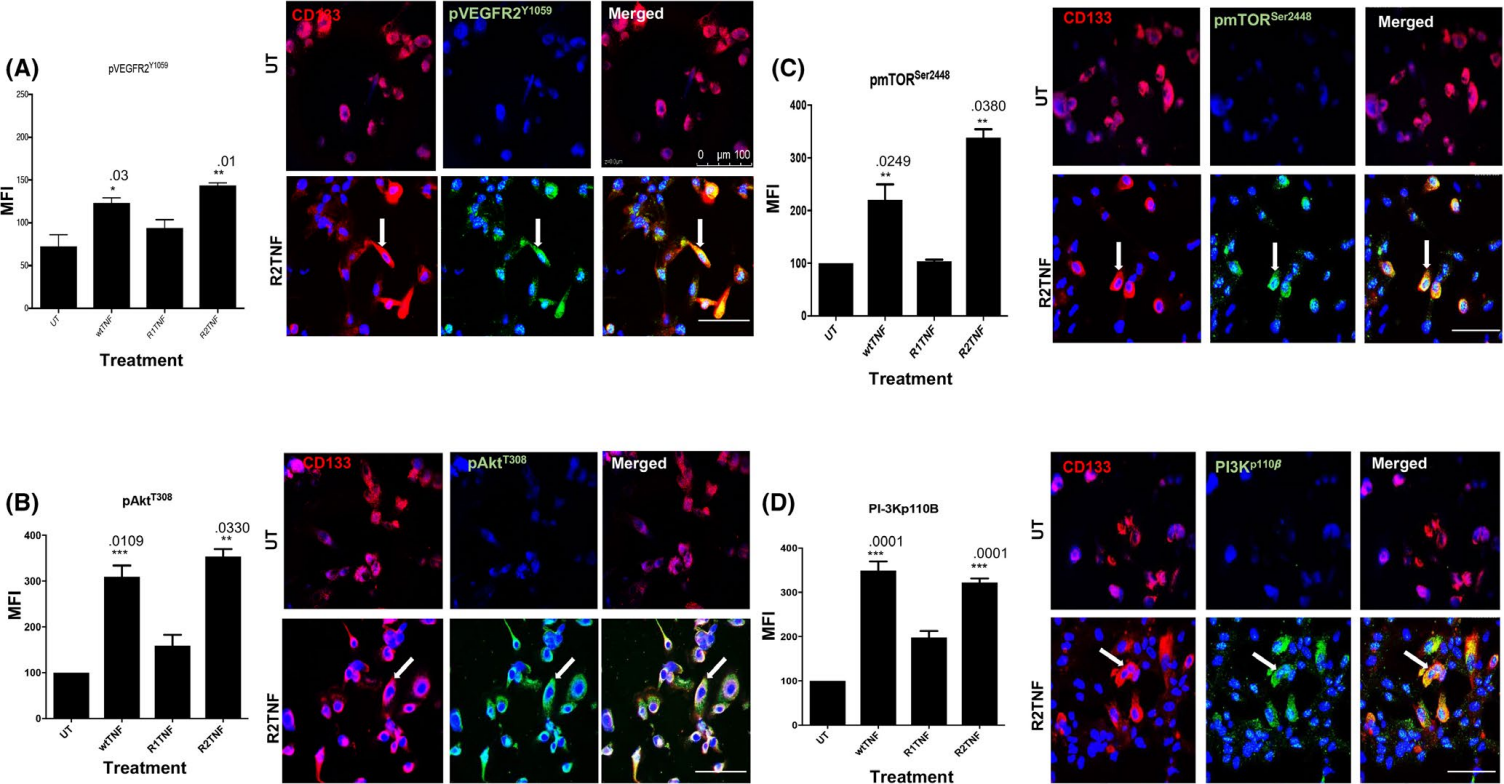
Isolates of ccRCC‐^CD133+^CSCs were treated with either wild type‐(wt)TNF, R1TNF or R2TNF or left untreated (UT‐in media alone) for 30 min at 37°C than immunostained for phosphorylated VEGFR2^Y1059^, PI‐3K^p110β^, Akt^Thr308^, and mTORC^Seri2448^. Flow cytometry analysis presented as mean fluorescence intensity (MFI) shows wtTNF and R2TNF (not R1TNF) induction of phosphorylation of all the four kinases as compared to UT controls and immunoreactive cells (a*rrows)* are illustrated in representative confocal images (A‐D). Blue nuclei stained with Hoechst 33342. Paired Student's *t* test. Error bars represent mean ± SEM N = 3 independent experiments of three different isolates with similar results. One way ANOVA. Mag ×63, Scale bars: 100 μmol/L

**Figure 4:**
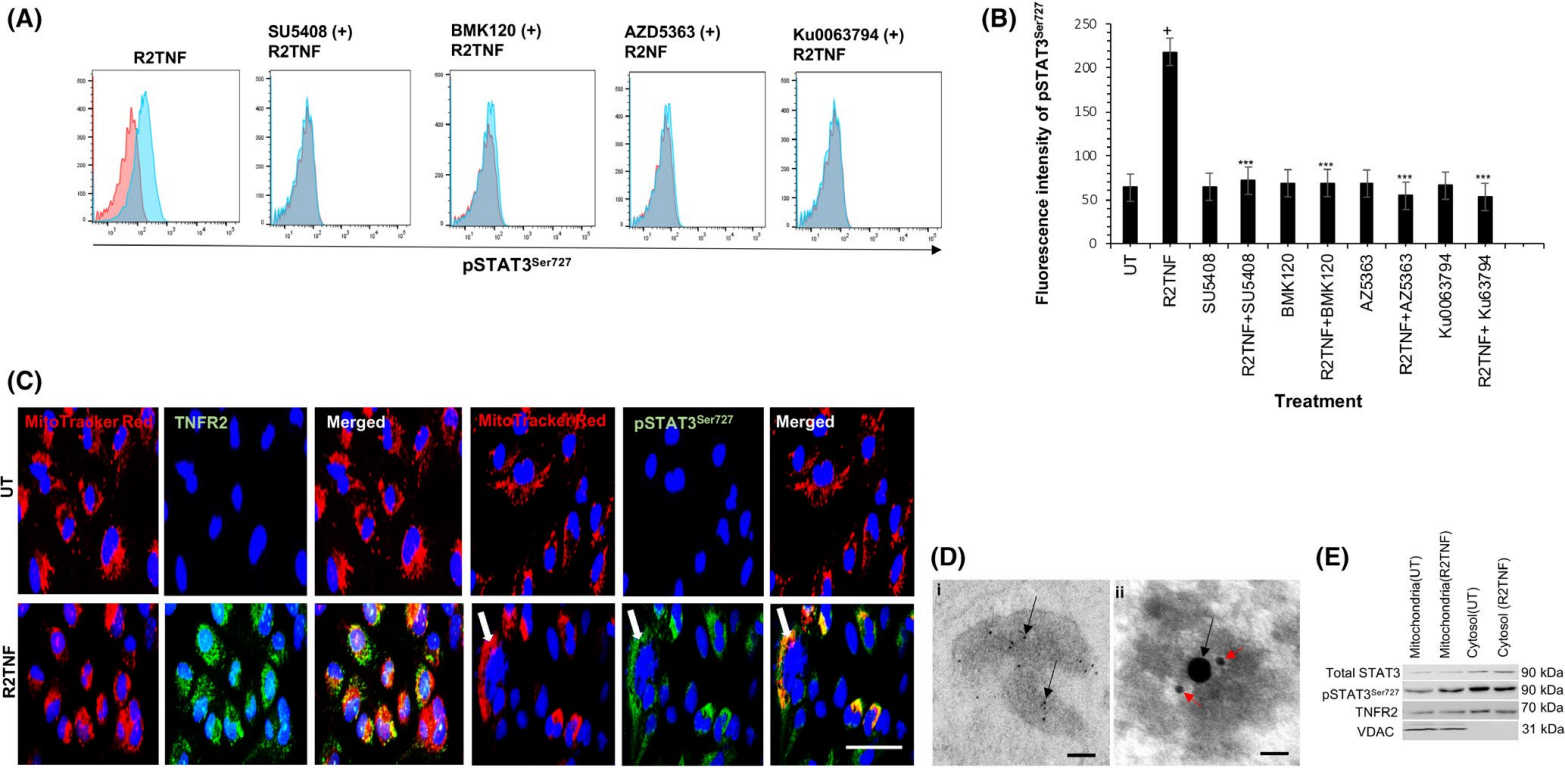
Isolates of ccRCC‐^CD133+^CSCs were treated with either R2TNF or vehicle alone (DMSO, marked as UT) for 30 min at 37°C or pretreated for 1h with specific inhibitors to VEGFR2 (SU5408‐1 μmol/L), PI‐3K (BMK120‐4 μmol/L), Akt (AZ5363‐0.8 μmol/L), and mTORC1/2 (Ku0063794‐5 μmol/L) prior to R2TNF. A, Flow cytometry analysis shows the R2TNF induction of pSTAT3^Ser727^ (blue peaks) as compared to UT controls (red peaks), diminished by the inhibitors, and quantified in (B). Error bars represent mean ± SEM; ^+^ *P* < .0001––vs UT and ^***^ *P* < .0001––vs R2TNF. C, ccRCC‐^CD133+^CSCs incubated with MitotrackerRED for 15 min at 37°C prior to R2TNF shows the induction of TNFR2 and pSTAT3^Ser727^ expression in mitochondria (*arrows*) as compared to UT controls. D, Mitochondrial fraction from R2TNF‐treated cultures subjected to immuno‐gold labeling confirm mitochondria using antibody to voltage‐dependent anion channel (VDAC) conjugated to 5 nm‐gold (panel *i*) (*arrows*) and presence of TNFR2 (*20nm‐gold*)(*black arrows*) and pSTAT3^Ser727^ (*5nm‐gold*) (*red arrows*) (panel *ii*). E, Representative immunoblot of cytosolic and mitochondrial fraction from UT and R2TNF‐treated RCC‐immortalized cell line (*RCC‐26*) shows TNFR2 and pSTAT3^Ser727^ expression in both fractions. Blue nuclei stained with Hoechst 33342. N = 3 independent experiments of three different isolates with similar results. One way ANOVA. Mag ×63, Scale bars: C = 100 μmol/L, D = 200 nm

Our previous studies^3^ revealed that an important step in the TNFR2 signaling pathway in ccRCC linked to cell‐cycle entry involves cross‐talk between phosphorylation of VEGFR2 at tyrosine‐1059 (pVEGFR2^Y1059^) and cytosolic protein tyrosine kinase Etk. Phosphorylated VEGFR2 then initiates a signaling cascade leading to the activation of Akt. In other cell types where this pathway has been studied in detail, the first step involves the phosphorylation of a class 1 PI‐3K on its 110β subunit (PI‐3K^p110β^). This activates the PI‐3K p85 catalytic subunit, which phosphorylates phosphatidylinositol 4,5 bis‐phosphate (PIP2) to form phosphatidylinositol 3,4,5 tris‐phosphate (PIP3) in the inner leaflet of the plasma membrane. The generation of PIP3 promotes the binding of both phosphoinositide‐dependent kinase (PDK)‐1 and Akt to the plasma membrane inner leaflet and when brought into proximity, PDK‐1 partially activates Akt through threonine 308 phosphorylation (Akt^Thr308^). Complete activation of Akt requires a second phosphorylation event at serine 473 that can be mediated by several different kinases, including the mechanistic target of rapamycin mTOR as part of mTOR complex (mTORC)2. However, even partial activation of Akt can promote cell survival.^35^, ^36^, ^37^ Fully activated Akt activates mTORC1 by phosphorylating serine‐2448 (mToR^Ser2448^),^38^ which is linked to protein synthesis and cell growth. Flow cytometry and IF analysis confirmed that wtTNF and R2TNF (not R1TNF), induce pVEGFR2^Y1059^, Akt^Thr308^, mToR^Ser2448^, and PI‐3K^p110β^ in ccRCC‐^CD133+^ CSCs compared to UT controls (Figure 3A‐D). To determine the relevance of this pathway for TNFR2‐mediated pSTAT3^Ser727^ signaling, cells were pretreated with specific inhibitors to these kinases––SU5408, BMK120, AZ5363 or Ku0063794 (VEGFR2, PI‐3K, Akt, and mTOR, respectively) and each decreased R2TNF‐induced pSTAT3^Ser727^ formation by ~4‐fold in ccRCC‐^CD133+^CSCs (Figure 4A, quantified in B). These data indicate a role for the ETK/VEGFR2/PI‐3K/Akt/mTORC pathway in TNFR2‐mediated pSTAT3^Ser727^ in ccRCC‐^CD133+^CSCs.

### 3.3 Inhibition of VEGFR2/PI‐3K/Akt/mTORC pathway induced cell death in ccRCC‐^CD133+^CSCs

To confirm the involvement of the VEGFR2/PI‐3K/Akt/mTORC pathway in TNFR2‐mediated pSTAT3^Ser727^ in ccRCC‐^CD133+^CSCs, we assessed the effect of specific inhibitors to these kinases either alone or in combination with wtTNF, R1TNF or R2TNF. Cells were either left UT or pretreated with the inhibitors alone for 1h or with inhibitors prior to TNF treatment for 30min (37°C) and subjected to TUNEL. The percentage of cell death was calculated using the formula: TUNEL‐^positive cells^/total cells (×100) at ×40 Mag. wtTNF and R1TNF induced cell death (Table 1). In the absence of inhibitors, R2TNF also induced cell death, but to a significantly lesser extent than wtTNF or R1TNF as previously reported.^4^ Each of the kinase inhibitors alone also induced cell death and this was only slightly increased by the addition of wtTNF, R1TNF or R2TNF. These data further suggest that the VEGFR2/PI‐3K/Akt/mTORC pathway may protect from cell death.

**Table 1:** Quantification of the percentage of TUNEL^‐positive^ isolates of ccRCC‐^CD133+^CSCs calculated as [TUNEL^‐positive^/total cells (×100)] at ×40 magnification

Treatment	UT (%)	wtTNF (%)	R1TNF (%)	R2TNF (%)
UT	3.60 ± 1.0	35.9 ± 2.0^***^	32.6 ± 0.6^***^	7.30 ± 1.4^*^
SU5408	59.3 ± 0.1^+^	65.2 ± 0.8^+^	63.5 ± 0.9^+^	60.1 ± 0.1^+^
BMK120	57.6 ± 0.6^+^	59.6 ± 0.5^+^	58.26 ± 0.2^+^	52.2 ± 0.1^+^
AZ5363	57.2 ± 0.9^+^	62.3 ± 0.7^+^	69.7 ± 0.8^+^	61.7 ± 0.2^+^
Ku00063794	57.9 ± 0.3^+^	63.2 ± 0.4^+^	61.2 ± 0.3^+^	60.2 ± 0.3^+^

Note

Cells were either treated with a vehicle DMSO (UT) or treated with wild‐type (wt)TNF, R1TNF or R2TNF alone for 30 min or treated with specific kinase inhibitors alone for 1h or pretreated with inhibitors prior to TNF for 30min (37°C). Values represented as mean ± SEM. ^*^ *P* < .05,^***^ *P* < .001, ^+^ *P* < .0001 vs UT. N = 3 independent experiments of 3 different isolates with similar results.

Abbreviations: AZD5363, Akt inhibitor‐0.8 μmol/L; BMK120, Buparlisib, PI‐3K inhibitor‐4 μmol/L; DMSO, Dimethyl sulfoxide; Ku0063794, mTORC1/2 inhibitor‐5 μmol/L; R1TNF and R2TNF, muteins selectively binds to TNFR1 or TNFR2‐1 μg/mL; SU5408, VEGFR2 inhibitor‐1 μmol/L; Wild‐type TNF, (wt)TNF‐10 ng/mL.

### 3.4 wtTNF and R2TNF induce the mitochondrial localization of TNFR2 and pSTAT3^Ser727^ in ccRCC‐^CD133+^CSCs

While pSTAT3^Ty705^ is known to translocate to and act in the nucleus as a transcription factor, pSTAT3^Ser727^ exerts biological effects in mitochondria.^16^, ^18^, ^19^, ^20^ Since ligation of TNFR2 increases the expression of TNFR2 and pSTAT3^Ser727^, we wondered if these proteins are expressed and associated with mitochondria in ccRCC‐^CD133+^CSCs. To test this, cells treated with R2TNF for 30 minutes were postincubated with MitoTracker™Red for 15 minutes (37°C) and subjected to IF using the antibody to TNFR2 or pSTAT3^Ser727^. In comparison to UT controls, which showed a rare signal, R2TNF induced a marked signal for TNFR2 or pSTAT3^Ser727^ expression in mitochondria in ccRCC‐^CD133+^CSCs (Figure 4C), also detected by IGEM (Figure 4D). As we were unable to obtain a sufficient number of ccRCC‐^CD133+^CSCs for immunoblotting isolated mitochondria, we isolated mitochondria from a ccRCC immortalized cell line (RCC‐26) treated with R2TNF or left UT and observed that R2TNF induced the expression of TNFR2 and pSTAT3^Ser727^ (Figure 4E). These data suggest that R2TNF mediates increased expression and translocation of both TNFR2 and pSTAT3^Ser727^ to mitochondria in ccRCC‐^CD133+^CSCs.

### 3.5 siRNA knockdown of TNFR2 and STAT3 reduce cell viability and increase cell death in ccRCC‐^CD133+^CSCs

**Figure 5:**
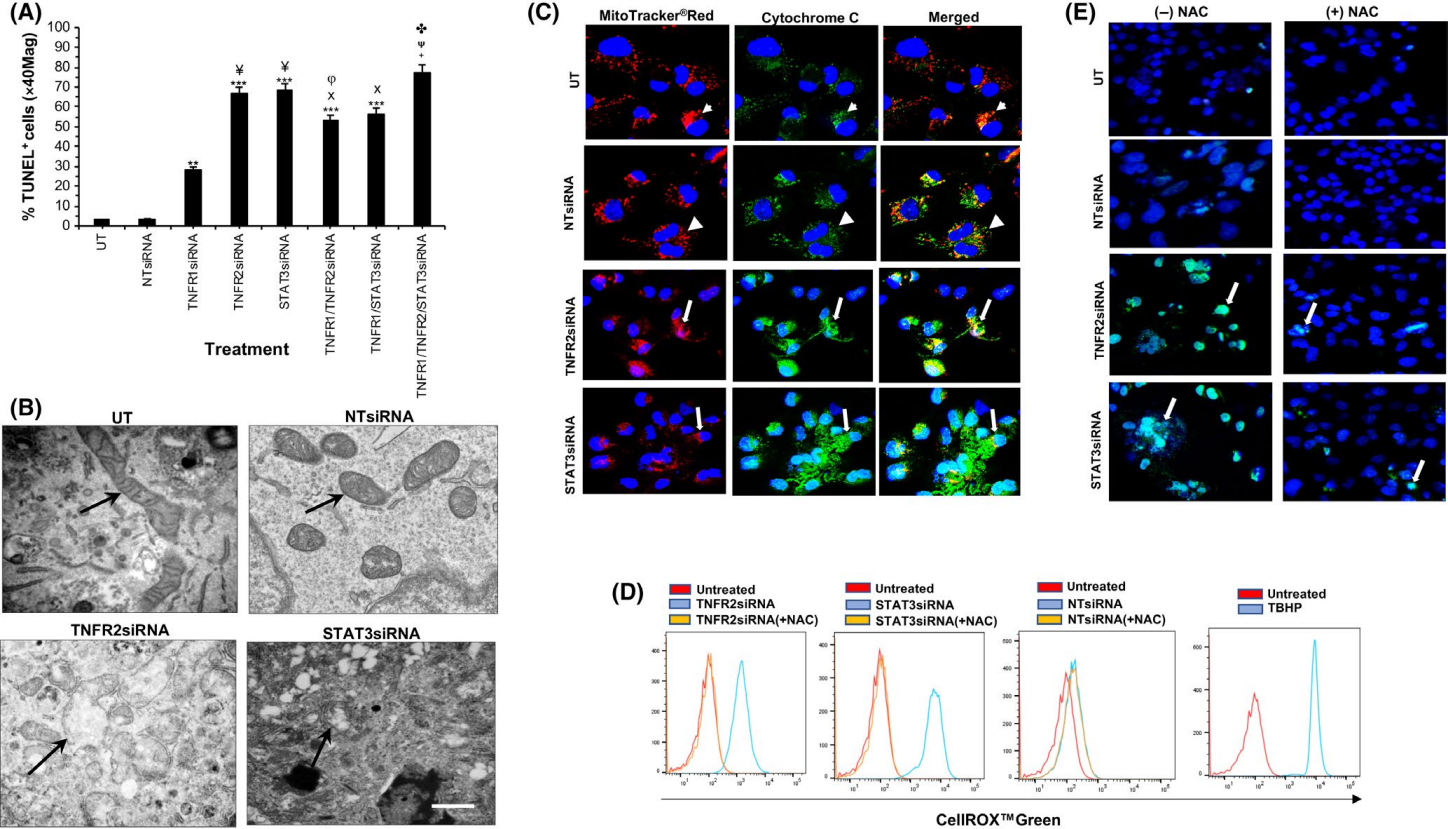
(A) Isolates of ccRCC‐^CD133+^CSCs were transfected with siRNAtargeting TNFR1 for 24h or TNFR2 or STAT3 for 72 h at 37°C or with control siRNA (nontargeting siRNA‐NTsiRNA or left untreated‐UT‐in media alone) shows increased cell death (presented as percentage of TUNEL‐positive cell/total cells (×100) at ×40 Mag) in the absence of all three proteins, more pronounced in the absence of TNFR2 or STAT3 signals as compared to absence of TNFR1 or control group. Notably, the absence of TNFR1 signals in combination with the absence of TNFR2 or STAT3 signals confer protection by a small margin. Error bars represent mean ± SEM; ^**^ *P* < .01 or ^***^ *P* < .001‐ vs control group, ^¥^ *P* < .0001‐ vs TNFR1siRNA; ^φ^P < 0.001‐ vs TNFR2siRNA or STAT3siRNA or TNFR1/TNFR2/STAT3siRNA; ^X^P < 0.0001‐vs TNFR2siRNA or STAT3siRNA; ^Ψ^P < 0.001‐vs TNFR1siRNA; ^✤^ *P* < .01‐ vs TNFR1/or TNFR2 and/or STAT3siRNA. B, Electron micrographs show loss of mitochondria cristae and distorted outer membrane in the absence of TNFR2/STAT3 signals as compared to controls (*arrows*) and cytochrome c release in cytosol (*small arrows*) (C) as compared to define and punctate staining in controls (*arrowheads*) illustrated by confocal microscopy. D, Flow cytometry analysis demonstrates the induction of CellROX™Green expression (Blue) in the absence of TNFR2/STAT3 signals, diminished by anti‐oxidant N‐Acetyl Cysteine (NAC) (Orange). UT controls (Red); positive control (ROS inducer Tert‐butyl hydroperoxide‐TBHP) (Blue). E, Representative confocal images of similar cultures show the nuclear signal for CellROX™Green diminished by NAC (*arrows*). Blue nuclei stained with Hoechst 33342. N = 3 independent experiments of three different isolates with similar results. Mag ×63, One way ANOVA. Scale bars: C and E = 100 μmol/L, B = 500nM

To determine if TNFR2 and pSTAT3^Ser727^ are linked to cell viability in ccRCC‐^CD133+^CSCs, TNFR2 and STAT3 protein expression were knocked down using siRNA. Representative immunoblot shows ~80%‐90% knockdown efficiency at 72h posttransfection (Figure S1A). The effect of knockdown expression on cell viability was analyzed by CCK‐8, TBED, and TUNEL. CCK‐8 demonstrated significant suppression in cell viability in the absence of TNFR2 signals (41%, 13%, 10%) and STAT3 signals (37%, 17%, 12%) compared with control groups (UT and NTsiRNA––99%, 98%, 99%), more pronounced at 48 and 72h (^+^ *P* < .0001) as compared to 24h (^***^ *P* < .001), respectively. Treatment with R2TNF alone showed minimal effect on cell viability (95%, 81%, 85%) compared to control groups and did not show a significant effect in the absence of TNFR2 signals (40%, 15%, 15%), but did induce a minor protection in the absence of STAT3 signals (47%, 34%, 31%). wtTNF or R1TNF alone also caused a significant reduction in cell viability (76%, 49%, 33%, and 75%, 43%, 40%) but this effect was much less than that induced by the absence of TNFR2/STAT3 signals. Treatment with wtTNF in the absence of TNFR2/STAT3 signals caused only a small further reduction in cell viability (Figure S1B). TBED showed similar findings (data not shown). We wondered if death in these circumstances was mediated by unopposed autocrine/paracrine TNF signaling via TNFR1. To address the role of TNFR1 more directly, cells were transfected with siRNA targeting TNFR1^39^ at the same time as siRNA targeting TNFR2 and/or STAT3 for 48 hours, followed by treatment with wtTNF for 30 minutes (37°C). Immunoblot analysis demonstrated low levels of TNFR1 expression in control groups (UT and NTsiRNA), which was slightly induced by wtTNF than further diminished by TNFR1siRNA (Figure S1C). Unexpectedly, the absence of TNFR1 signals induced some degree of cell death (detected by TUNEL) (quantified in Figure 5A) but significantly less than that induced by the absence of TNFR2 or STAT3 signals either alone (^¥^ *P* < .0001) or in combination (^φ^ *P* < 0.001). The absence of all three signals induced an even higher level of cell death (^✤^ *P* < .01). Some protection was conferred with the knockdown expression of TNFR1 in the absence of either TNFR2 or STAT3 signals alone (^x^ *P* < 0.0001) but not in combination (^Ψ^ *P* < .001). The degree of cell death induced by the absence of TNFR1, TNFR2, and STAT3 either alone or in combination compared to control groups is illustrated in phase contrast micrographs (Figure S1D). Collectively, these data indicate that cell death induced by the absence of TNFR2 and of STAT3 signals are largely independent of TNFR1 signals.

### 3.6 Mitochondria promote cell death induced by the absence of TNFR2 and of STAT3 signals in ccRCC‐^CD133+^CSCs

Since TNFR2 signaling causes both TNFR2 and pSTAT3^Ser727^ to localize to mitochondria in ccRCC‐^CD133+^CSCs, we examined if mitochondria played a role in cell death when these signals were disrupted. We first evaluated the effect of TNFR2 and STAT3 knockdown expression on mitochondrial morphology using TEM of mitochondrial fractions. In comparison to the control groups, the absence of TNFR2/STAT3 signals induced noticeable changes in mitochondrial morphology with loss of cristae and a discontinuous outer membrane (Figure 5B). Mitochondria promote cell death through two general mechanisms, which may not be mutually exclusive. The first involves the loss of mitochondrial outer membrane integrity resulting in the escape of certain mitochondrial proteins into the cytosol. The other is mediated by pathological increases in ROS generation through dysregulation of the mitochondrial ETC*.* 40, 41 Excessive ROS production may lead to apoptosis or necroptosis. We used surrogate assays to detect these events. To determine the loss of mitochondrial outer membrane integrity, we assessed the cytochrome c release following the knockdown expression of TNFR2 or STAT3 in ccRCC‐^CD133+^CSCs. Cells were incubated with MitoTracker™Red for 15 minutes (37°C) and immunostained using anti‐cytochrome c. In comparison to control groups (UT and NTsiRNA), which showed a strong punctate staining of cytochrome c in mitochondria, the absence of TNFR2/STAT3 signals resulted in a diffuse intense signal in the cytoplasm (Figure 5C). These data indicate that mitochondrial release of proteins into the cytosol may contribute to cell death induced by the absence of TNFR2/STAT3 signals. We were unable to perform subcellular fractionation for the detection of cytochrome c following siRNA transfection of ccRCC‐^CD133+^CSCs due to insufficient cell number. Next, we evaluated changes in cellular ROS levels using the oxidative stress‐sensitive dye CellROX Green by flow cytometry and IF‐confocal microscopy. Flow cytometry showed a strong signal in the absence of TNFR2/STAT3 signals compared to control groups, significantly diminished by ROS scavenger NAC (Figure 5D). IF‐confocal microscopy demonstrated a marked signal in cells with fragmented nuclei and distorted outer membrane, significantly less with NAC pretreatment by ~7‐fold in the absence of TNFR2 signals and by ~5‐fold in the absence of STAT3 signals as compared to the control group (Figure 5E). The addition of wtTNF did not induce any significant changes.

### 3.7 TNFR2/STAT3 signals protect ccRCC‐^CD133+^CSCs from cell death mediated predominantly through a caspase‐independent necroptotic pathway

To determine the cell death mechanism induced by the absence of TNFR2/STAT3 signals, we assessed the number of cells containing cleaved caspase 3 (a marker of apoptotic cell death) and serine‐358 phosphorylation of mixed lineage kinase domain‐like pseudokinase (pMLKL^Ser358^, a marker of necroptotic cell death). Following siRNA targeting TNFR2 and STAT3 in ccRCC‐^CD133+^CSCs, dying cells identified by TUNEL were analyzed by IF using antibody to cleaved caspase‐3^p175^ and pMLKL^Ser358 2^. Of the 85.2% ± 0.9% TUNEL^+^cells, only 18.9% ± 0.4% were positive for cleaved caspase‐3^p175^ in the absence of TNFR2 signals and 12.5% ± 0.9% in the absence of STAT3 signals. The addition of wtTNF in the absence of TNFR2 or STAT3 signals increased cell death by 21.4% ± 0.2% and 13.3% ± 0.3%. The addition of R1TNF showed similar findings to wtTNF. wtTNF or R1TNF alone also induced the expression of cleaved caspase‐3^p175^, but significantly less (9.5% ± 0.2% and 8.3% ± 0.5%). R2TNF induced minimal effect (2.1% ± 0.1%). In comparison, 65.8% ± 0.2% was positive for pMLKL^Ser358^ in the absence of TNFR2 signals and 70.2% ± 0.4% in the absence of STAT3 signals slightly increased by wtTNF (^+^TNFR2siRNA 66.1% ± 0.5% and ^+^STAT3siRNA 75.1% ± 1.4%). wtTNF or R1TNF alone induced pMLKL^Ser358^ but significantly less (16.8% ± 1.2% and 18.2% ± 0.4%).

Next, we utilized specific inhibitors to confirm the involvement of cleaved caspase‐3^p175^ and pMLKL^Ser358^ on cell death induced by the absence of TNFR2/STAT3 signals. Cultures were treated with z‐VAD.fmk (a pan‐caspase inhibitor) or necrostatin‐1 (Nec‐1; an inhibitor of RIPK1 kinase activity),^2^ either alone or in combination, or prior to siRNA targeting TNFR2 and STAT3 (±wtTNF) and cell death quantified by TUNEL. zVAD.fmk conferred partial protection ~2‐fold from cell death induced by the absence of TNFR2/STAT3 signals, more attenuated by Nec‐1 ~ 5‐fold, with an additive effect with a combination of the inhibitors ~7‐fold (Figure S2A,B). The addition of wtTNF did not induce an additive effect. These data imply that TNFR2 and STAT3 signals are important in sustaining cell viability and that their absence induces cell death predominantly via a caspase‐independent pathway in ccRCC‐^CD133+^CSCs, consistent with our previous report.^21^

### 3.8 Comparison between CD133^+^ cells from ccRCC and NK

In a final series of experiments, we compared responses of CD133^+^ CSCs in ccRCC and normal kidney stem cells (NK‐^CD133+^cells) in organ cultures and their isolated counterparts. As shown in Table 2, NK‐^CD133+^cells show similar but less marked responses to wtTNF and TNFR2. Resident NK‐^CD133+^cells in organ cultures induced significantly less expression of pSTAT3^Ser727^ (~2‐fold) in response to wtTNF and R2TNF as compared to in CD133^+^CSCs in cRCC organ cultures (Figure S3A,B). Moreover, the number of cells that responded to TNFR2 by colocalization of TNFR2 and pSTAT3^Ser727^ were significantly less (~15%) as compared to CD133^+^CSCs in ccRCC organ cultures (~35%) (Figure S3C,D). Like their counterparts, isolated NK‐^CD133+^ cells expressed stem‐cell markers (Figure S3E,F) and responded to TNFR2 by the induction of pSTAT3^Ser727^ (Figure S4A‐D) and its colocalization with TNFR2 but the percentage of immunoreactive cells was significantly less (~8% vs ~70% in ccRCC‐^CD133+^CSCs) (Figure 4E, quantified in 4F). However, in contrast to ccRCC‐^CD133+^CSCs, activation of the kinase cascade appears to predominantly involve Akt and mTOR, with less activation of VEGFR2 and PI3K (Figure S5A‐D) and specific inhibition of Akt and mTOR resulted in a diminished R2TNF‐mediated pSTAT3^Ser727^ induction (Figure S6A,B) and cell death but significantly less (~2‐fold) compared to that observed in ccRCC‐^CD133+^CSCs (Table S1). Ligation of TNFR2 also induces the mitochondrial translocation of TNFR2 and STAT3 in NK‐^CD133+^cells (Figure S6C).

**Table 2:** Comparison between CD133^+^ cells in short term organ cultures and isolates from normal kidney (NK) and clear cell renal carcinoma (ccRCC)

NK organ cultures				ccRCC organ cultures		
% Immuno‐positive cells						
Treatment	pSTAT3^Ser727^	pSTAT3^Ty705^	TNFR2/pSTAT3^Ser727^	pSTAT3^Ser727^	pSTAT3^Ty705^	TNFR2/pSTAT3^Ser727^
UT	1.0 ± 0.1	1.0 ± 0.2	1.0 ± 0.1	6.0 ± 0.2	1.0 ± 0.2	3.2 ± 0.1
wtTNF	13 ± 0.2	1.0 ± 0.2	15.0±0.1	46 ± 0.4	1.0 ± 0.2	35 ± 0.6
R1TNF	2.0 ± 0.5	1.0 ± 0.4	3.0 ± 0.1	7.0 ± 0.2	1.0 ± 0.3	3.1 ± 0.2
R2TNF	12.1 ± 0.1	1.0 ± 0.3	13.1 ± 0.2	44.1 ± 0.2	1.0 ± 0.2	33.1 ± 0.3

NK‐^CD133+^cells				ccRCC‐^CD133+^CSCs		
Median fluorescence intensity			Immuno‐positive cells	Median Fluorescence Intensity		Immuno‐positive cells
	pSTAT3^Ser727^	pSTAT3^Ty705^	TNFR2/pSTAT3^Ser727^	pSTAT3^Ser727^	pSTAT3^Ty705^	TNFR2/pSTAT3^Ser727^
UT	100	100	1.0 ± 0.1	100	100	1.5 ± 0.1
wtTNF	201 ± 2.0	101 ± 0.3	8.1 ± 0.5	695 ± 0.3	104.1 ± 0.5	70.2 ± 0.3
R1TNF	87.2 ± 0.1	101 ± 0.1	1.0 ± 0.3	115 ± 1.2	100.5 ± 0.1	3.3 ± 0.4
R2TNF	219 ± 0.8	101 ± 0.1	7.5 ± 0.1	772 ± 1.4	102.7 ± 0.2	68.3 ± 0.2

Median fluorescence intensity								
	pVEGFR2^pY1059^	PI‐3K^p110β^	pAkt‐^Thr308^	pmTORC^Ser2448^	pVEGFR2^pY1059^	PI‐3K^p110β^	pAkt‐^Thr308^	pmTORC^Ser2448^
UT	100	100	100	100	100	100	100	100
wtTNF	101	104 ± 0.1	150 ± 0.3	210 ± 0.2	236 ± 2.6	350 ± 0.5	310 ± 0.7	330 ± 1.5
R1TNF	102	100	110 ± 0.3	110 ± 0.1	103 ± 0.1	101 ± 0.1	110 ± 0.2	101 ± 0.1
R2TNF	105	106 ± 0.2	220 ± 1.8	230 ± 0.1	231 ± 1.5	330 ± 1.7	380 ± 0.9	350 ± 0.8

Note

Wild‐type (wt)TNF and R2TNF (but not R1TNF) induce pSTAT3^Ser727^, but not pSTAT3^Ty705^ and, colocalization of TNFR2 and pSTAT3^Ser727^. wtTNF and R2TNF‐mediated pSTAT3^Ser727^ occurs via the activation of VEGFR2, PI3‐K, Akt, and mTORC as specific inhibition of these kinases result in a diminished expression of TNF‐mediated pSTAT3^Ser727^, more pronounced in CD133^+^cells from ccRCC as compared to NK.

Percentage represents mean ± SEM, n = 3 samples and each treatment group.

Abbreviations: NTsiRNA, nontargeting siRNA; pAkt^Thr308^, phosphorylated Protein B at Threonine 308; PI‐3Kp100β, phosphorylated phosphoinositide 3‐kinases at 110β; pmTORC^Ser2448^, phosphorylated mammalian Target of Rapamycin at serine 2448; pSTAT3^Ser727^, phosphorylated STAT3 serine 727; pSTAT3^Ty705^, phosphorylated STAT3 tyrosine 705; pVEGFR2^Y1059^, phosphorylated‐vascular growth factor receptor 2 at Tyrosine 1059; R1TNF and R2TNF, mutein selectively binds to TNFR1 or TNFR2 −1 μg/mL; STAT3siRNA, siRNA targeting STAT3; TNFR2siRNA, siRNA targeting TNFR2; UT, untreated ‐ in media alone; Wild type, (wt)TNF −10 ng/mL.

The absence of TNFR2/STAT3 signals in NK‐^CD133+^cells also suppressed cell viability and induced cell death but significantly less than in ccRCC‐^CD133+^CSCs (~3‐fold vs ~9‐fold) (Figure S7A,B). Knockdown of TNFR1 signals also induced a small degree of cell death in NK‐^CD133+^cells and as in ccRCC‐^CD133+^CSCs, absence of all three signals induced an even higher level of cell death but significantly less(~2‐fold) than in ccRCC‐^CD133+^CSCs (Figure S7C). Similarly, the majority of TUNEL^+^ cells were positive for pMLKL^Ser358^ (Figure S8A, quantified in Table 3), with cell death significantly attenuated by Nec‐1, more so by its combination with z‐VAD.fmk (quantified in Table 4). Cell death in NK‐^CD133+^cells was also associated with cytochrome c release in the cytosol and generation of ROS but the frequency/intensity of these factors was notably less than in ccRCC‐^CD133+^CSCs (Figures S8B‐D, quantified in Table 5). In summary, NK‐^CD133+^cells show similar but less marked responses to TNFR2. The percentage of NK‐^CD133+^cells that respond by colocalization of TNFR2 and pSTAT3^Ser727^ is significantly less and activation of the kinase cascade appears to predominantly involve Akt and mTOR, with less activation of VEGFR2 and PI‐3K than in ccRCC‐^CD133+^CSCs (Figure 2). Ligation of TNFR2 also induces mitochondrial translocation of STAT3 in NK‐^CD133+^cells, but the propensity of these cells to undergo mitochondrial‐mediated necroptosis is significantly reduced and inhibiting this response results in less cell death.

**Table 3:** Quantification of percentage of TUNEL^+^CD133^+^cells from normal kidney (NK‐^CD133+^cells) and clear cell renal carcinoma (ccRCC‐^CD133+^CSCs) alone or in combination with immunostaining for pMLKL^Ser358^ or Clv‐Casp3^p175^ following treatment with wtTNF, R1TNF, or R2TNF (30 min/37°C) or with siRNA targeting TNFR2, STAT3, or negative controls (UT and NTsiRNA) at 72h (37°C)

Treatment	NK‐^CD133+^Cells (%)			ccRCC‐^CD133+^CSCs (%)		
	TUNEL^+^	Clv‐Casp3^p175+^	pMLKL^Ser358+^	TUNEL^+^	Clv‐Casp3 ^p175+^	pMLKL^Ser358+^
UT	0.1 ± 0.1	0.1 ± 0.1	0.1 ± 0.1	3.0 ± 0.2	0.1 ± 0.1	0.1 ± 0.1
wtTNF	19.2 ± 0.2	6.2 ± 0.5	15.3 ± 0.2	25.3 ± 0.3	9.5 ± 0.2	16.8 + 1.2
R1TNF	22 ± 0.3	5.2 ± 0.1	15.9 ± 0.4	26.2 ± 0.1	8.3 ± 0.5	18.2 ± 0.4
R2TNF	2.0 ± 0.1	1.9 ± 0.3	1.2 ± 0.1	3.2 ± 0.2	2.1 ± 0.1	1.9 ± 0.2
NTsiRNA	1.5 ± 0.5	0.1 ± 0.1	0.1 ± 0.1	3.2 ± 0.3	0.1 ± 0.1	0.1 ± 0.1
NTsiRNA (+T)	19.5 ± 0.2	5.8 ± 0.4	14.9 ± 0.7	24.8 ± 1.2	8.9 ± 1.1	15.2 ± 0.3
TNFR2siRNA	65.1 ± 0.4	11.9 ± 0.3	52.9 ± 0.1	85.2 ± 0.9	18.9 ± 0.4	65.8 ± 0.2
TNFR2siRNA (+T)	68.5 ± 0.7	12.3 ± 0.2	10.8 ± 0.3	87.1 ± 1.5	21.4 ± 0.2	66.1 ± 0.5
STAT33siRNA	60.1 ± 0.4	10.8 ± 0.2	49.6 ± 0.7	85.2 ± 0.5	12.5 ± 0.9	70.2 ± 0.4
STAT3siRNA (+T)	63.2 ± 0.2	9.8 ± 0.1	53.2 ± 0.4	86.3 ± 1.2	13.3 ± 0.3	75.1 ± 1.4

Note

wtTNF and R1TNF alone (but not R2TNF) induce cell death but by at smaller margin as compared to the absence of TNFR2 or STAT3 signals. No additive effect is seen with addition of wtTNF in the absence of TNFR2 or STAT3 signals. A higher number of TUNEL^+^ cells are positive for pMLKL^Ser358^ as compared to Clv‐Casp3^p175^

Percentage represents mean ± SEM, n = 3 samples and each treatment group.

Abbreviations: Clv‐Casp3^p175^, cleaved caspase3^p175^; NTsiRNA, nontargeting siRNA; pMLKL^Ser358^, phosphorylated mixed like kinase like domain at Serine358; R1TNF and R2TNF, wtTNF mutein selectively binds to TNFR1 or TNFR2 – 1 μg/mL; STAT3siRNA, siRNA targeting STAT3; TNFR2siRNA, siRNA targeting TNFR2; UT, Untreated, in media alone (1 h/37°C), T‐wtTNF (10 ng/mL)

**Table 4:** Quantification of the percentage of TUNEL^+^CD133^+^cells from normal kidney (NK‐^CD133+^cells) and clear cell renal carcinoma (ccRCC‐^CD133+^CSCs) following treatment with wtTNF, R1TNF or R2TNF alone for 30 min/37°C or with siRNA targeting TNFR2 or STAT3 or negative controls (UT and NTsiRNA) for 72 h/37°C (±wtTNF) and (±z‐VAD‐fmk and/or necrostatin‐1) for 1 h/37°C prior to treatment

Treatment	NK‐^CD133+^Cells (%)				ccRCC‐^CD133+^CSCs (%)			
	TUNEL^+^	+z	+n	+z/+n	TUNEL^+^	+z	+n	+z/+n
UT	0.1 ± 0.1	0.1 ± 0.1	0.1 ± 0.1	0.1 ± 0.1	3.0 ± 0.2	0.7 ± 0.1	0.5 ± 0.1	0.1 ± 0.1
wtTNF	19.2 ± 0.2	9.6 ± 0.1	5.3 + 0.1	3.8 ± 0.2	25.3 ± 1.3	5.1 ± 0.1	3.42 ± 0.1	2.4 ± 0.2
R1TNF	22.5 ± 0.7	11.9 ± 0.1	4.8 ± 0.4	3.3 ± 0.1	26.2 ± 0.7	5.9 ± 0.3	6.2 ± 0.4	2.3 ± 0.1
R2TNF	2.0 ± 0.9	1.1 ± 0.1	1.0 ± 0.4	1.0 ± 1.0	3.2 ± 0.9	1.2 ± 0.3	0.2 ± 0.4	1.0 ± 0.1
NTsiRNA	1.0 ± 0.1	0.2 ± 0.1	1.1 ± 0.2	0.1 ± 0.1	2.1 ± 0.1	0.6 ± 0.4	2.2 ± 0.2	0.3 ± 0.2
NTsiRNA (+T)	18.5 ± 0.7	8.9 ± 0.5	4.8 ± 0.2	2.9 ± 0.3	24.1 ± 0.8	5.6 ± 0.4	3.6 ± 0.2	2.8 ± 0.2
TNFR2siRNA	65.2 ± 0.3	32.1 ± 0.1	12.8 ± 0.3	9.8 ± 0.8	85.2 ± 0.9	26.0 ± 0.4	13.9 + 0.3	10.9 ± 0.2
TNFR2siRNA (+T)	68.3 ± 0.2	34.3 ± 0.1	13.6 ± 0.1	9.8 ± 0.9	87.1 ± 0.3	39.1 ± 0.2	14.8 ± 0.1	12.6 ± 1.1
STAT3siRNA	60.1 ± 0.1	30.2 ± 0.1	12.2 ± 0.4	8.5 ± 0.3	83.2 ± 0.2	25.0 ± 0.1	12.2 ± 0.4	9.2 ± 0.2
STAT3siRNA (+T)	63.2 ± 0.1	31.5 ± 0.6	12.4 ± 0.5	9.6 ± 02	86.3 ± 0.2	42.1 ± 0.7	13.6 ± 0.5	10.7 ± 1.2

Note

wtTNF and R1TNF alone induce cell death, more pronounced with the absence of TNFR2 or STAT3 signals. Pretreatment with z‐VAD‐fmk diminishes cell death, attenuated by necrostatin‐1 and further by a combination of the two and, significantly more in ccRCC‐^CD133+^CSCs CSCs than in NK‐^CD133+^cells.

Percentage represents mean ± SEM, n = 3 samples and each treatment group.

Abbreviations: n, necrostatin‐1 (30 μmol/L); NTsiRNA, nontargeting siRNA; R1TNF and R2TNF, wtTNF mutein selectively binds to TNFR1 or TNFR2 ‐ 1 μg/mL; STAT3siRNA, siRNA targeting STAT3; TNFR2siRNA, siRNA targeting TNFR2; UT, Untreated (in media alone), T‐Wild‐type (wt)TNF (10 ng/mL); z, z‐VAD.fmk (20 μmol/L)

**Table 5:** Quantification of flowcytometry (top) and immunofluorescence data (bottom) of CD133^+^cells from normal kidney (NK‐^CD133+^cells) and clear cell renal carcinoma (ccRCC‐^CD133+^CSCs) positive for CellROX*®* Green (marker of ROS generation) following siRNA targeting TNFR2 or STAT3 or negative controls (UT and NTsiRNA) for 72h/37°C or for immunostaining data treatment with wtTNF, R1TNF or R2TNF alone for 30min/37°C or post‐treatment with wtTNF after siRNA transfection (±NAC, ROS scavenger) for 1h/37°C

Treatment	NK‐^CD133+^ cells		ccRCC‐^CD133+^CSCs	
	Median fluorescence intensity (CellROX® Green)			
	(‐) NAC	(+) NAC	(‐) NAC	(+) NAC
UT	100	100	100	100
NTsiRNA	101.3 ± 0.8	100.3 ± 0.3	103.9 ± 0.2	104.2 ± 0.2
TNFR2siRNA	1231.3 ± 11.2	87.1 ± 0.5	2320.3 ± 10.6	90.1 ± 0.5
STAT3siRNA	2046.3 ± 7.2	92.4 ± 0.3	5653.6 ± 1.4	90.4 ± 0.5

% of CellROX ® green ‐positive cells				
	(‐) NAC	(+) NAC	(‐) NAC	(+) NAC
UT	0.1 ± 0.1	0.1 ± 0.1	0.3 ± 0.2	0.3 ± 0.1
wtTNF	14.1 + 2.1	3.2 ± 1.2	32.0 ± 1.3	5.7 ± 1.0
R1TNF	13.9 ± 0.9	2.6 ± 1.2	17.8 ± 0.9	5.0 ± 0.2
R2TNF	2.1 ± 0.6	1.7 ± 0.1	3.5 ± 0.3	1.2 ± 0.1
NTsiRNA	0.1 ± 0.1	0.1 ± 0.1	0.9 ± 0.1	0.1 ± 0.1
NTsiRNA (+T)	14.2 ± 0.7	3.0 ± 0.5	34.3 ± 1.2	5.3 ± 0.5
TNFR2siRNA	31.4 ± 0.3	9.1 ± 0.9	67.5 ± 0.2	10.2 ± 1.9
TNFR2siRNA (+T)	34.0 ± 1.2	9.3 ± 0.5	68.7 ± 1.6	11.1 ± 1.0
STAT3siRNA	22.1 ± 1.2	7.3 ± 0.7	56.2 ± 1.2	14.1 ± 0.2
STAT3siRNA (+T)	25.1 ± 1.9	7.1 ± 0.4	67.9 ± 1.2	15.1 ± 0.7

Note

CellROX Green staining is increased after wtTNF or R1TNF treatment, further pronounced by the absence of TNFR2 or STAT3 signals and significantly diminished by NAC.

Percentage represents mean ± SEM, n = 3 samples and each treatment group.

Abbreviations: NAC, N‐Acetyl Cysteine (anti‐oxidant‐250 mmol/L); NTsiRNA, nontargeting siRNA; R1TNF and R2TNF, wtTNF mutein selectively binds to TNFR1 or TNFR2 – 1 μg/mL; STAT3siRNA, siRNA targeting STAT3; TNFR2siRNA, siRNA targeting TNFR2; UT, Untreated (in media alone); T‐Wild‐type (wt)TNF (10 ng/mL).

## 4 DISCUSSION

We previously reported that ligation of TNFR2 drives ccRCC‐^CD133+^CSCs proliferation and increases their sensitivity to cell cycle‐dependent cytotoxicity.^21^ Here we elucidate the mechanism of the growth‐promoting effect of TNFR2 signaling in CD133^+^CSCs in ccRCC using molecular, cellular, and imaging techniques. We demonstrate various important findings: (a) TNF rapidly induces (within 30 minutes) pSTAT3^Ser727^, but not pSTAT3^Ty705^ and its colocalization with TNFR2 in mitochondria, (b) TNF induces the phosphorylation of VEGFR2, PI‐3K, Akt, and mTORC1/2 and specific inhibition of these phosphorylated proteins effectively suppresses TNFR2‐med0iated pSTAT3^Ser727^ expression and increases cell death, (c) the absence of TNFR2/STAT3 signals causes cell death, mitochondrial structural changes, associated with translocation of cytochrome c to the cytoplasm, activation of cleaved caspase‐3^p175^, phosphorylation of MLKL^Ser358^, and generation of high levels of ROS, (d) cell death induced by the absence of TNFR2/STAT3 signals is partially inhibited by z‐VAD.fmk, and further attenuated by Nec‐1, indicating that cell death is predominantly necroptotic, (e) wtTNF or R1TNF in the absence of TNFR2/STAT3 signals further increase cell death by a small margin, and (f) blocking TNFR1 signals does not confer absolute protection from cell death induced by the absence of TNFR2/STAT3 signals.

Although outcomes for patients with advanced RCC have improved in recent years with the development of targeted therapies, curative treatment remains elusive. The putative anti‐tumor activity of infliximab (anti‐TNF antibody) was demonstrated in advanced RCC,^42^ but a subsequent phase I/II trial using a combination of infliximab and the multi‐kinase inhibitor sorafenib showed adverse events with lack of efficacy.^43^ Our data indicate that blocking TNFR2 may account for adverse outcomes and suggest that a TNFR2 agonist combined with standard chemotherapy regimens may offer greater efficacy.

Within the tumor microenvironment, a complex network of signals affects tumor initiation and progression. TNF is a putative autocrine and paracrine growth factor in RCC^3^, ^44^ and increases stemness via the induction of epithelial‐mesenchymal transition.^45^ TNF and STAT3 can regulate pluripotency.^46^, ^47^, ^48^, ^49^ Moreover, TNF can mediate the induction of TNFR2 expression through STAT3 contributing to the tumor‐promoting roles of STAT3^50^ and regulate STAT3 through its binding to a −1,578 STAT response element in the TNFR2 promoter.^47^ These data support the role of STAT3 in TNFR2 signaling. We initially investigated whether TNF effects STAT3 phosphorylation in ccRCC‐^CD133+^ CSCs using a kidney tumor organ culture model and TNF receptor subtype‐selective muteins.^4^ Our data demonstrate that selective ligation of TNFR2 (not TNFR1) induce pSTAT3^Ser727^ but not pSTAT3^Ty705^ in resident CD133^+^CSCs in ccRCC. To study resident CD133^+^CSCs in detail we isolated them together with CD133^+^cells from nontumor zones for comparative studies. Similar to in situ CD133^+^cells, selective ligation of TNFR2 by R2TNF and wtTNF (but not R1TNF) induced pSTAT3^Ser727^ but not pSTAT3^Ty705^ in vitro in isolated CD133^+^cells from both ccRCC and NK, but the expression was more pronounced in ccRCC‐CD133^+^CSCs. Our data indicate that TNFR2‐mediated pSTAT3^Ser727^ involves the activation of VEGFR2/PI‐3K/Akt/mTORC1/2 pathway in ccRCC‐^CD133+^CSCs. As predicted, specific inhibitors of this pathway resulted in increased cell death, implicating them in the TNFR2/STAT3‐mediated survival pathway. The involvement of VEGFR2 in TNFR2 signaling is consistent with our previous report^3^ and further implicates the phosphorylation of VEGFR2 in TNFR2‐mediated survival.

The PI‐3K/Akt/mTOR pathway regulates cell survival in a variety of tumor types including RCC and aberrant hyperactivation is a target for cancer therapy.^51^ An association between suppression of PI‐3K/Akt/mTORC pathway and STAT3 on inhibition of cell proliferation has been reported.^52^ Collectively, our data suggest a close connection between TNFR2/pSTAT3^Ser727^ signaling and VEGFR2/PI‐3K/Akt/mTORC pathways in driving survival of ccRCC‐CD133^+^CSCs. These findings may explain why therapy directed against these kinases have provided only modest survival benefits.^53^ Moreover, clinical therapy involving adjuvant trial with receptor tyrosine kinases in RCC has also reported no improvement in disease‐free survival and patients have experienced adverse events.^54^ The importance of these kinases in driving the survival of CSCs via TNFR2 signaling and the ability of TNFR2 to sensitize CSCs to cytotoxic killing strongly suggest that a therapeutic strategy for the eradication of ccRCC would be cytotoxics in the absence of VEGFR2/PI‐3K/Akt/mTOR inhibition. However, further studies will be required to determine whether these kinases act in a cascade in response to TNFR2 signaling.

Our data showing TNFR2‐mediated mitochondrial expression of TNFR2 and its association with pSTAT3^Ser727^ implicate mitochondria in the survival pathway. Our EM data showing disrupted mitochondrial morphology in the absence of TNFR2/STAT3 signals further supports our line of thought. Previous studies have shown TNFR2 to confer mitochondria‐dependent cardioprotective effects via STAT3 activation*.* 55 We identified a TNF‐binding protein in the inner mitochondrial membrane that can be detected by a monoclonal antibody to TNFR2.^6^ The mechanism by which TNFR2 is recruited to the mitochondria is unclear. It may require an adaptor protein such as E3 ubiquitin ligase TRAF2 (TNF receptor‐associated factor‐2), which plays a key pro‐survival role as an adaptor protein to transduce the activation of kinases and transcription factors.^56^ Studies using rat pancreatic insulin‐producing INS‐1E beta cell lines have reported that TRAF2 is required for the IFNγ‐stimulated phosphorylation of STAT3 and that TRAF2 knockdown increases the cytokine‐induced production of ROS.^57^ An earlier report has detected the TNF‐induction of TRAF2 expression in mitochondria of RCC‐26 by immunoblotting.^58^ Thus, TRAF2 may be an important mediator of mitochondrial TNFR2/pSTAT3^Ser727^ signaling. Another member of the TNFR2 signaling complex associated with mitochondria is aminopeptidase P3 (APP3).^59^ Further studies will be required to determine the role of TNFR2 in mediating pSTAT3^Ser727^ localization in mitochondria and the role of TRAF2 and APP3 in TNFR2/STAT3 signaling in ccRCC‐^CD133+^CSCs. Alternatively, pSTAT3^Ser727^ may mediate its mitochondrial translocation.^60^ Indeed, STAT3 can act as a positive or negative regulator of mitochondrial activity depending on specific modifications.^61^ Mitochondrial pSTAT3^Ser727^ has been shown to be important in the growth/survival of cancer and its function is distinct from nuclear transcriptional regulation.^17^, ^62^ pSTAT3^Ser727^ but not pSTAT3^Ty705^ is crucial for the optimal activity of complexes I/II of the ETC regulating ROS concentrations and metabolic function.^17^, ^20^ Mitochondrial pSTAT3^Ser727^ has been shown to confer protection as a PS727‐STAT3 dominant negative eliminates protection against TNF‐induced mitochondrial stress and apoptosis.^63^ It is, therefore, reasonable to speculate that TNFR2 signaling may protect ccRCC‐^CD133+^CSCs by modulating STAT3 phosphorylation in mitochondria.

Using siRNA knockdown, we observed a dramatic increase in cell death with the absence of TNFR2/STAT3 signals altering mitochondrial morphology with the loss of cristae and outer membrane disruption, associated with cytosolic release of cytochrome c, generation of ROS, caspase‐3 cleavage, and MLKL phosphorylation. The specific mechanism of mitochondria in the induction of cell death in this context is unclear, but may involve ROS modulator 1 (Romo1) recruitment of B‐cell lymphoma‐extralarge (Bcl‐X(L).^64^ Whatever the mechanism, these data support a central role for mitochondria in TNFR2/STAT3 signaling. Additionally, increased expression of pMLKL^Ser358^ and attenuation of cell death by Nec‐1 as compared to z‐VAD.fmk strongly indicates the dominance of caspase‐independent cell death (necroptosis), consistent with our previous report.^2^ Maintenance of normal mitochondrial morphology has been shown to regulate several aspects of mitochondrial functioning, including mitochondrial metabolism, ROS production, and induction of apoptosis.^65^ Mitochondrial involvement in cell death involved cytochrome c release and combination with cytosolic apoptotic protease activating factor (APAF)‐1 and, in the presence of ATP, the formation of a complex that binds and promotes autocatalytic activation of the initiator caspase 9. Activated caspase 9 proteolytically activates executioner caspases including caspase‐3 resulting in apoptosis. Other released proteins, including second mitochondrial activator of cell death (SMAC)/Diablo, which augments caspase activation, or apoptosis inducing factor (AIF), may also trigger death.^66^ AIF is misnamed because, unlike cytochrome c and SMAC/Diablo, it acts independently of caspases^66^ and death may result from events triggered following its nuclear translocation and dysregulated mitochondrial function.

Importantly, we show that blocking TNFR1 signals confers only partial protection from cell death induced by the absence of TNFR2/STAT3 signals, suggesting the involvement of an unknown mechanism, perhaps similar to that reported by Lacerda et al, where TNF’s effect on mitochondria is independent of its cell surface receptors and occur via ROS generation.^50^ Unexpectedly, the absence of TNFR1 signals alone induced cytotoxic effect, albeit small compared to that induced by the absence of TNFR2/STAT3 signals either alone or in combination. wtTNF or R1TNF did not significantly attenuate cell death induced by the absence of TNFR2/STAT3 signals in ccRCC‐^CD133+^CSCs, indicating that they do not confer an additive effect. These data point to an essential role for TNFR2/STAT3 signaling in the survival of CSCs independent of TNFR1 signals. Although TNFR2 is regarded to have exclusively pro‐survival functions, whereas TNFR1 signals cell death,^2^ emerging evidence indicates some overlap in TNFR2 and TNFR1‐induced functions. Both TNF receptors are capable of mediating similar functions under certain circumstances, including cell death, proliferation, differentiation, and cytokine production.^67^ Various factors including cell type, cell activation state, intracellular or extracellular environment, and whether nuclear factor kappa‐B is constitutively expressed, may lead to a degree of functional overlap.^67^

**Figure 6:**
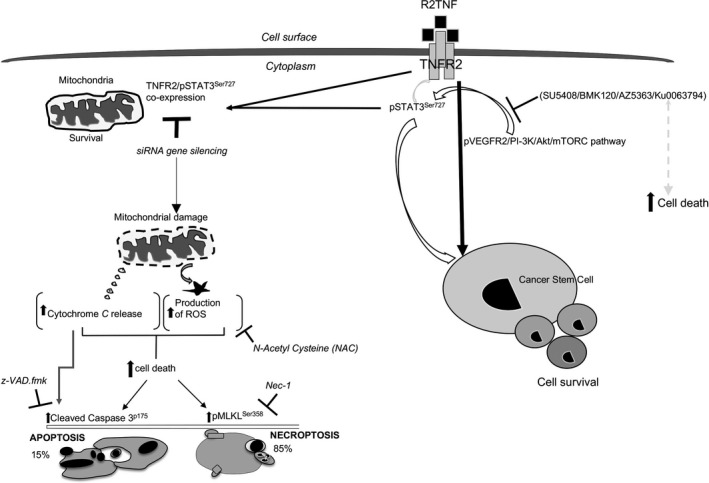
Schematic diagram of the consequences of TNFR2‐mediated survival signaling in ccRCC‐^CD133+^CSCs; selective ligation of TNFR2 mediates the induction of pSTAT3^Ser727^ through phosphorylation of VEGFR2/PI 3K/Akt/mTORC pathway leading to the survival of CSCs in ccRCC. Pharmacological inhibition of these phosphorylated kinases results in a diminished expression of TNF‐mediated pSTAT3^Ser727^ formation and causes an increase in cell death. Furthermore, selective ligation of TNFR2 mediates the expression of TNFR2 and its association with pSTAT3^Ser727^ in mitochondria. The absence of TNFR2/STAT3 causes an increase in cell death triggering change in mitochondrial ultrastructure, translocation of cytochrome c to cytosol, activation of cleaved caspase 3^p175^, phosphorylation of MLKL^Ser358^, and generation of ROS, which can be blocked by NAC. Cell death is partially inhibited by z‐VAD.fmk, further attenuated by Nec‐1, with an additive effect by a combination of both z‐VAD.fmk and Nec‐1, indicating the dominance of necroptotic cell death pathway

In summary, we propose the model shown in Figure 6: selective ligation of TNFR2 mediates the induction of pSTAT3^Ser727^ through the phosphorylation of VEGFR2/PI 3K/Akt/mTORC1/2 pathway leading to the survival of CSCs in ccRCC. Pharmacological inhibition of these phosphorylated kinases results in diminished expression of TNF‐mediated pSTAT3^Ser727^ formation and increases cell death. Furthermore, selective ligation of TNFR2 increases TNFR2 expression and its association with pSTAT3^Ser727^ in mitochondria. The absence of TNFR2/STAT3 increases cell death triggering changes in mitochondrial ultrastructure, release of cytochrome c, activation of cleaved caspase 3^p175^, phosphorylation of MLKL^Ser358^, and generation of ROS. Cell death is partially inhibited by z‐VAD.fmk, further attenuated by Nec‐1, with an additive effect of a combination of both z‐VAD.fmk and Nec‐1, indicating the dominance of necroptotic cell death.

The reported insensitivity of CSCs to chemotherapy and radiotherapy^68^ suggests that current anti‐cancer drugs, which inhibit tumor cells, may not effectively inhibit CSCs. The clinical relevance of targeting CSCs is supported by recent studies, including CD24 targeting for the treatment of pancreatic and colon,^69^ CD44 targeting for the treatment of acute myeloid leukemia^70^ and CD133 targeting for gastric and hepatocellular cancer.^71^ In conclusion, our data here and from our previous studies^21^ indicate that an alternative therapeutic strategy for eradication of ccRCC would be to promote CSCs survival by a TNFR2 selective agonist in combination with cell‐cycle dependent cytotoxic drugs. Importantly, mechanistic differences between normal stem cells and cancer stem cells could be targeted to deplete CSCs without damaging normal stem cells.

## CONFLICT OF INTEREST

The authors declare no competing interests.

## AUTHOR CONTRIBUTIONS

RAL, JP, and JRB designed the experiments. SP and AYW assisted with the recruitment of patients and acquisition of tissue. RAL carried out all experiments, data acquisition, and drafted the manuscript, SP, JSP, AYW, and JRB edited the manuscript. JW carried out the flow cytometry experiments. AYW provided pathological assessments.
