# Supplementary figures and images for: Tumor necrosis factor receptor‐2 signaling pathways promote survival of cancer stem‐like CD133+ cells in clear cell renal carcinoma
Source: FASEB Bioadv. 2020 Jan 3;2(2):126–44. doi: 10.1096/fba.2019-00071 (PMC7003657; doi:10.1096/fba.2019-00071)

Supplementary Figure 1.

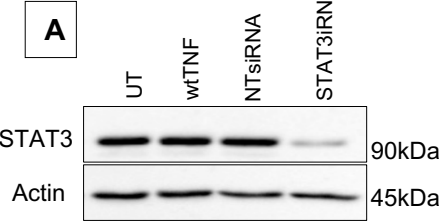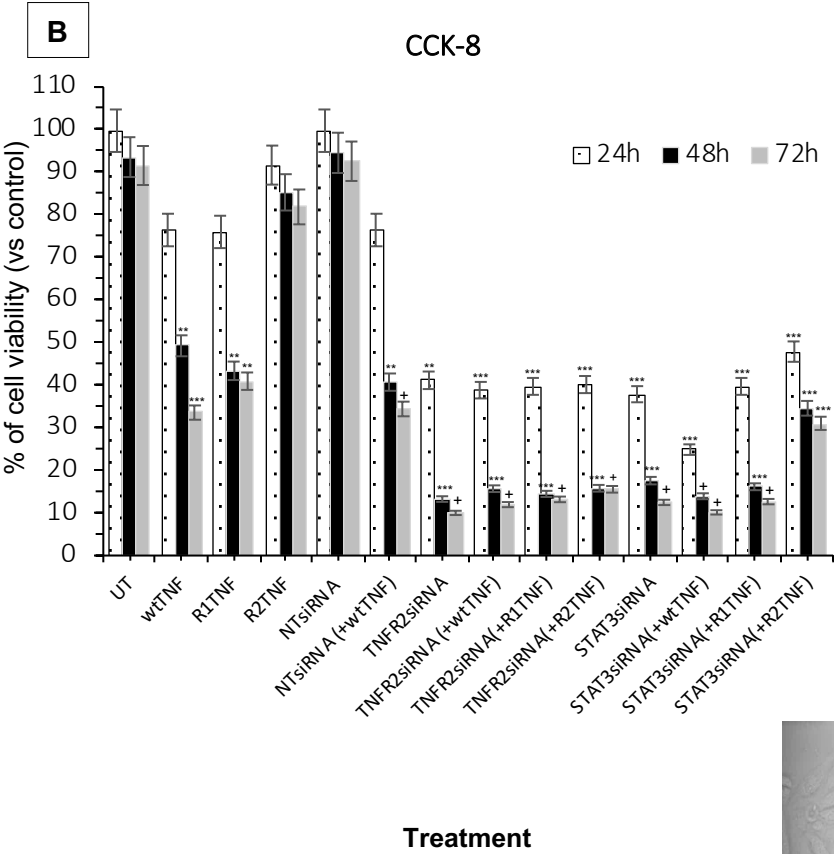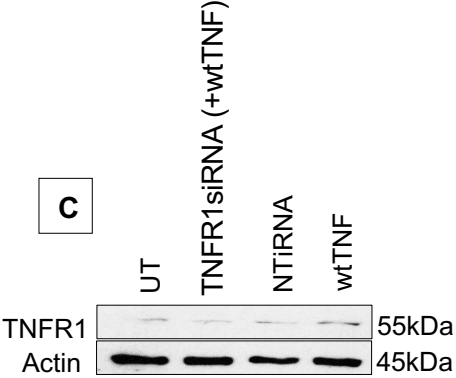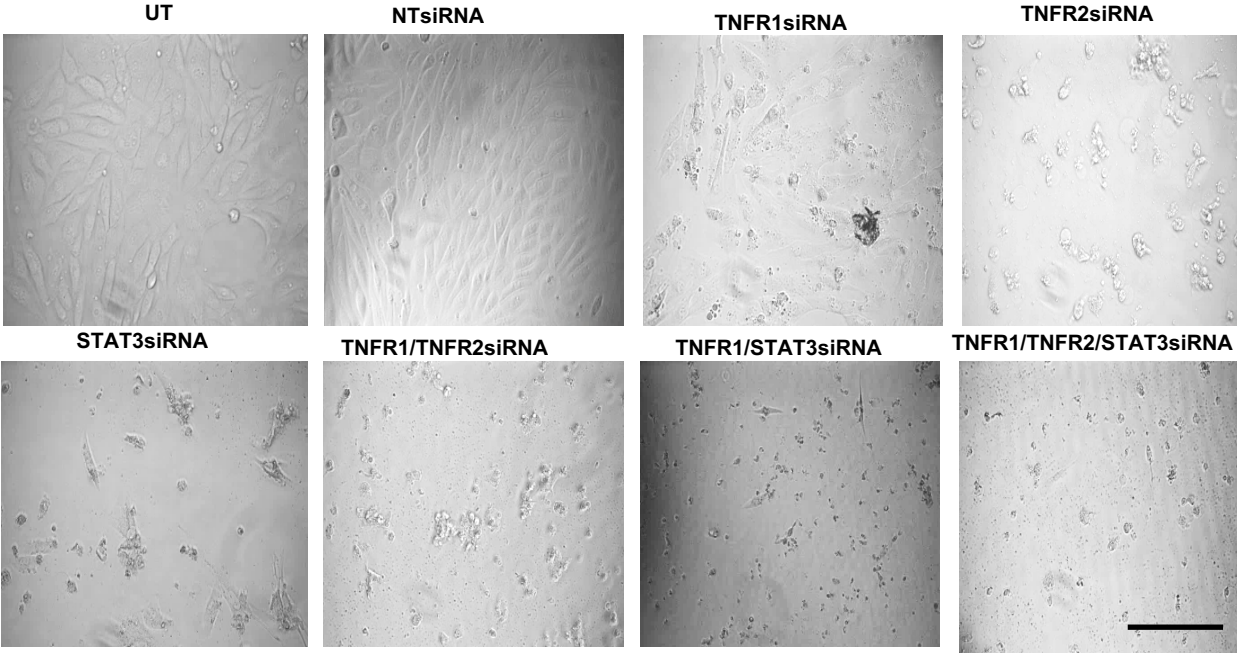

Supplement: Supplementary file 1 [file FBA2-2-126-s001.pdf]

Supplementary Figure 2.

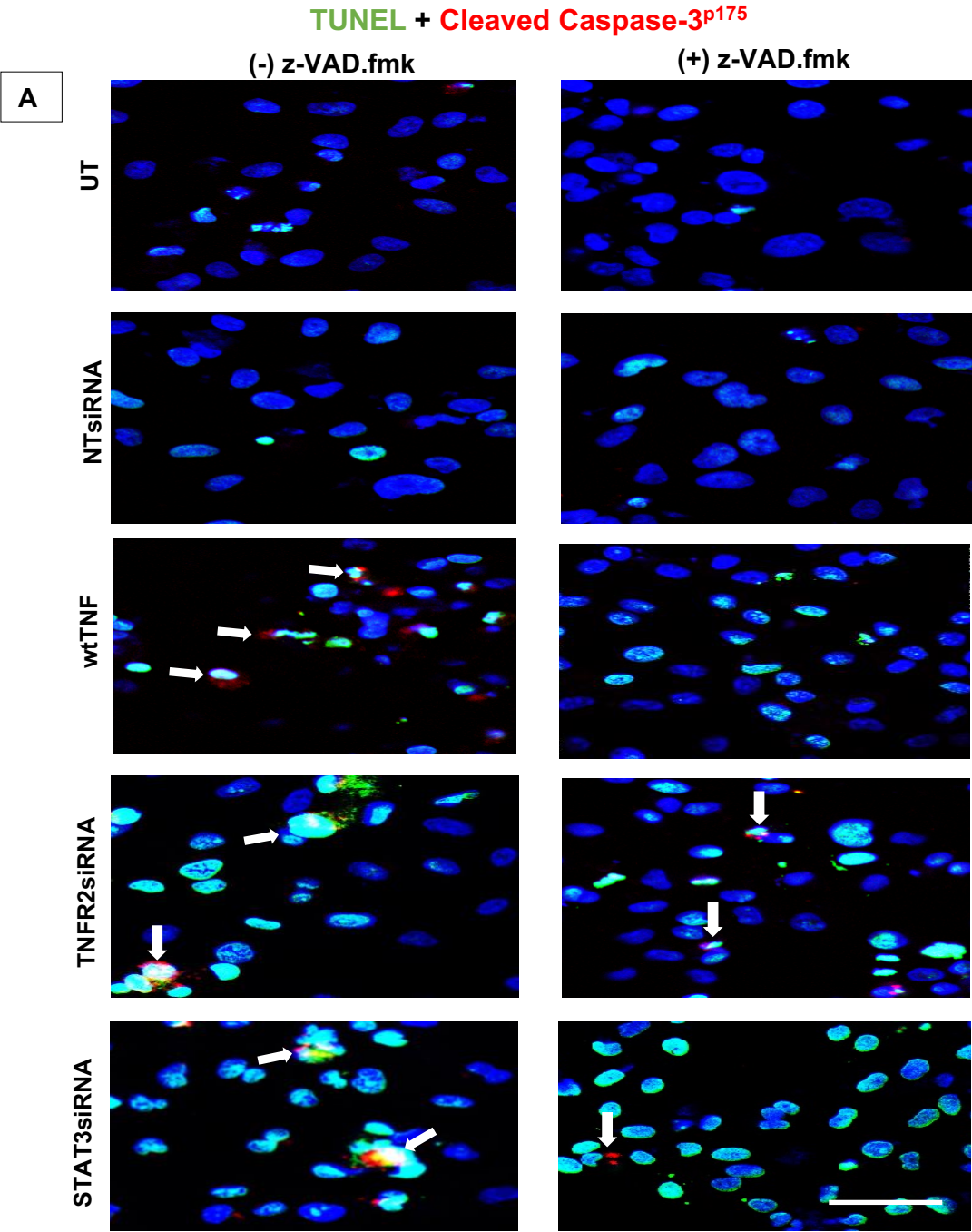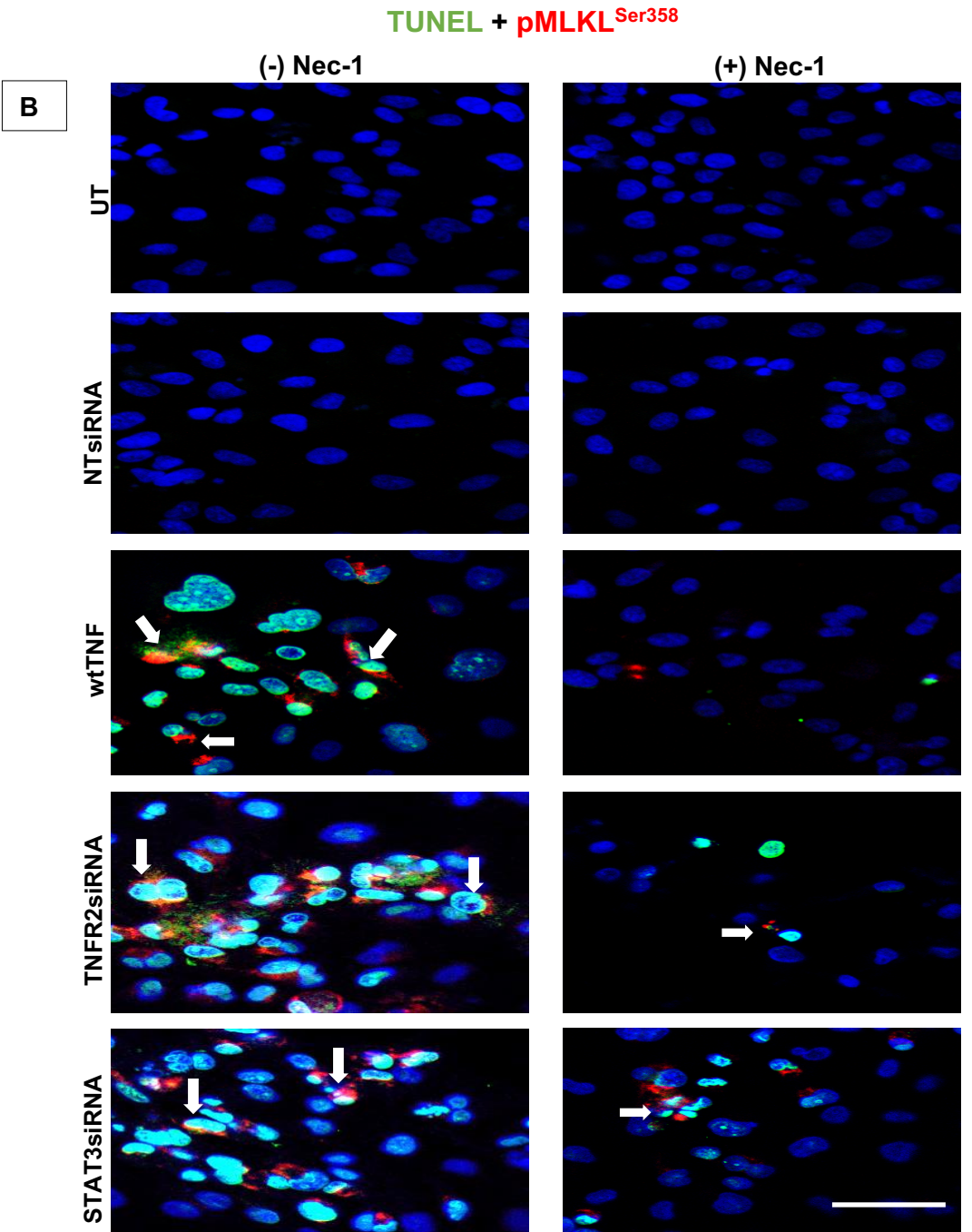

Supplement: Supplementary file 2 [file FBA2-2-126-s002.pdf]

Supplementary Figure 3.

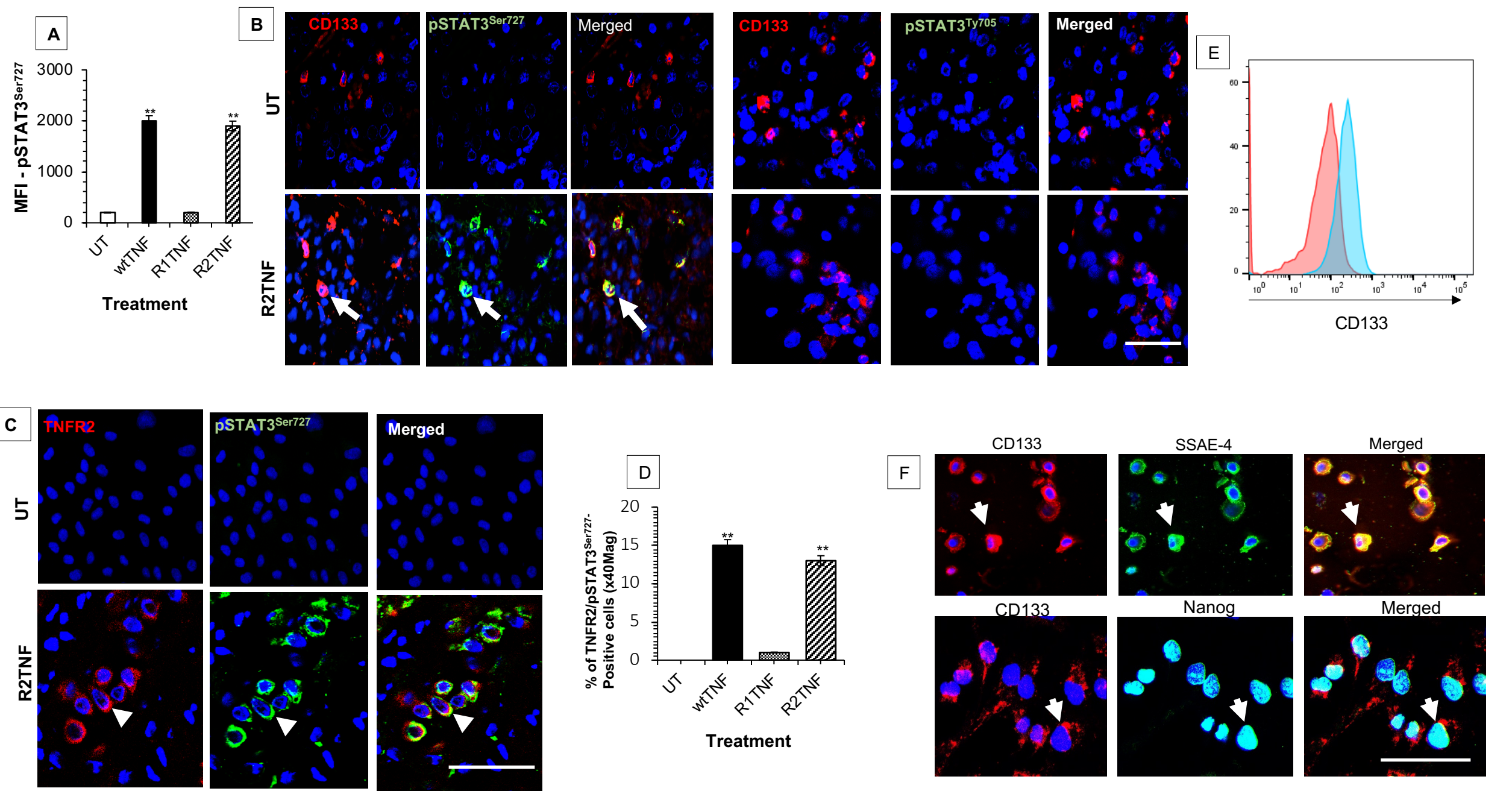

Supplement: Supplementary file 3 [file FBA2-2-126-s003.pdf]

Supplementary Figure 4.

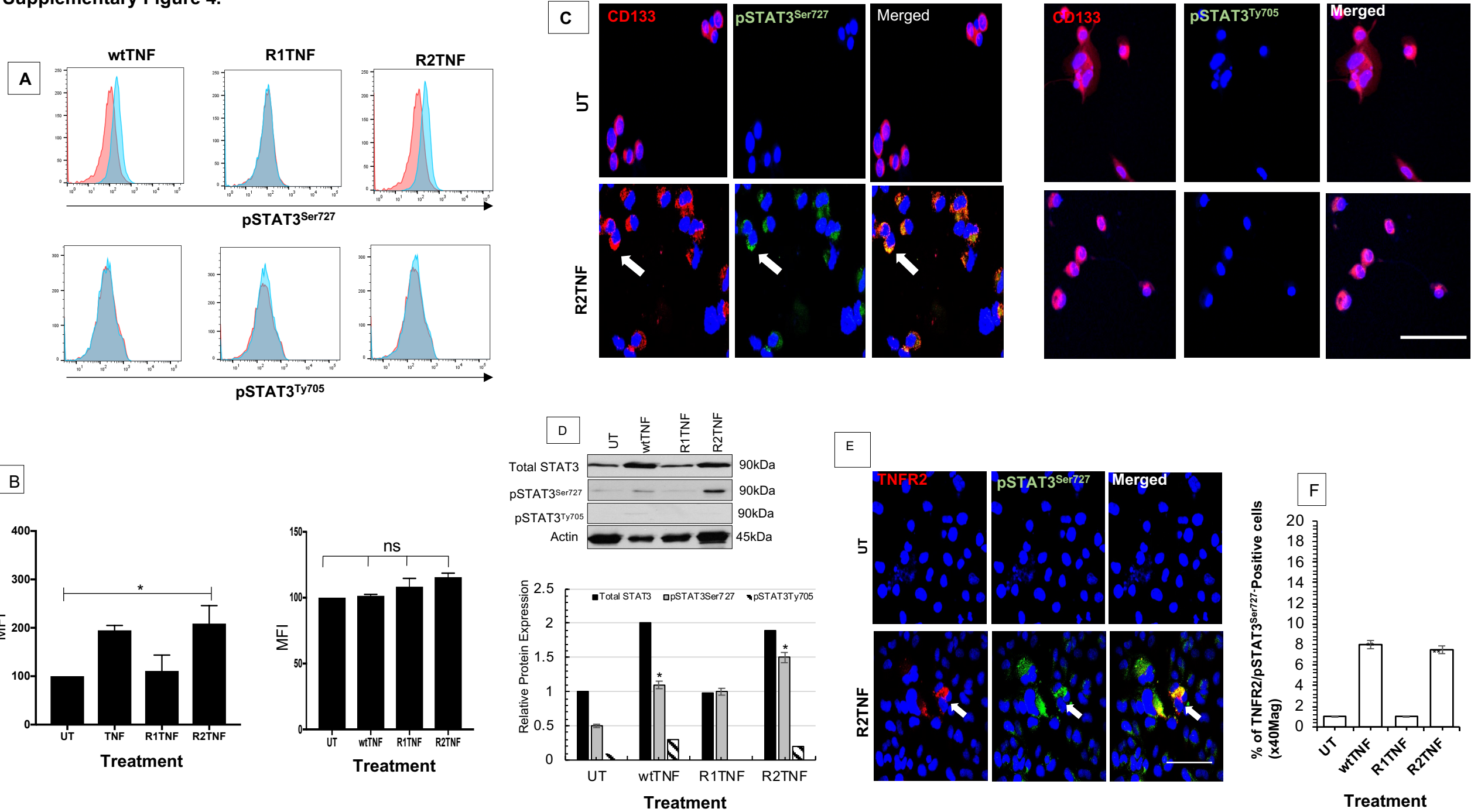

Supplement: Supplementary file 4 [file FBA2-2-126-s004.pdf]

**Supplementary Figure 5.**

**A**

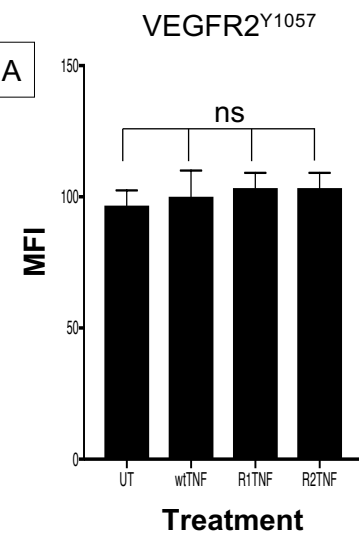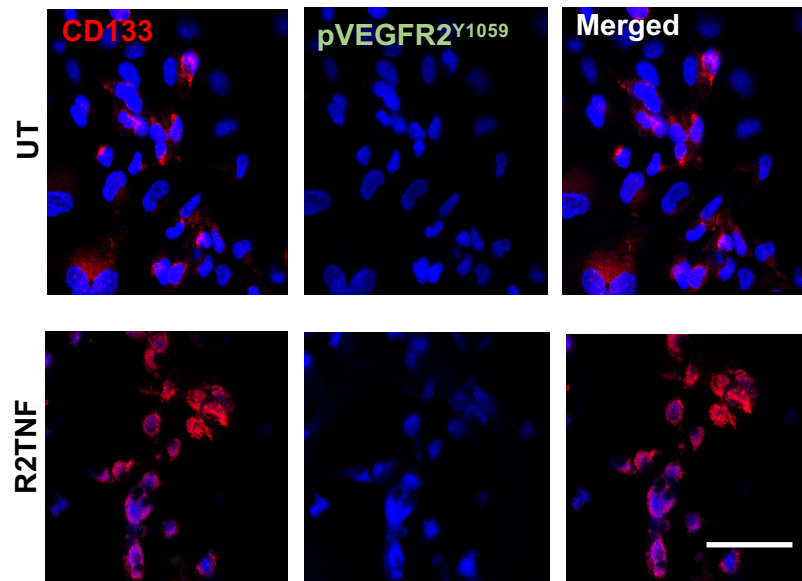

**C**

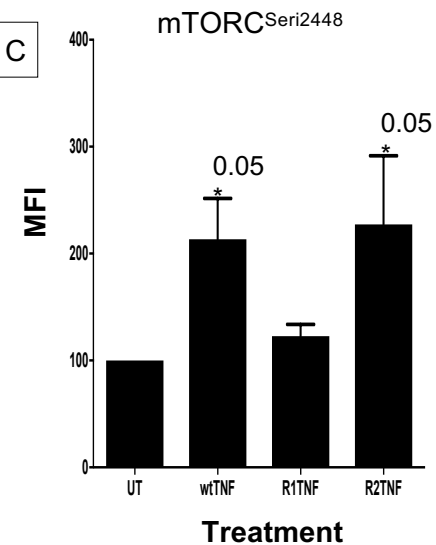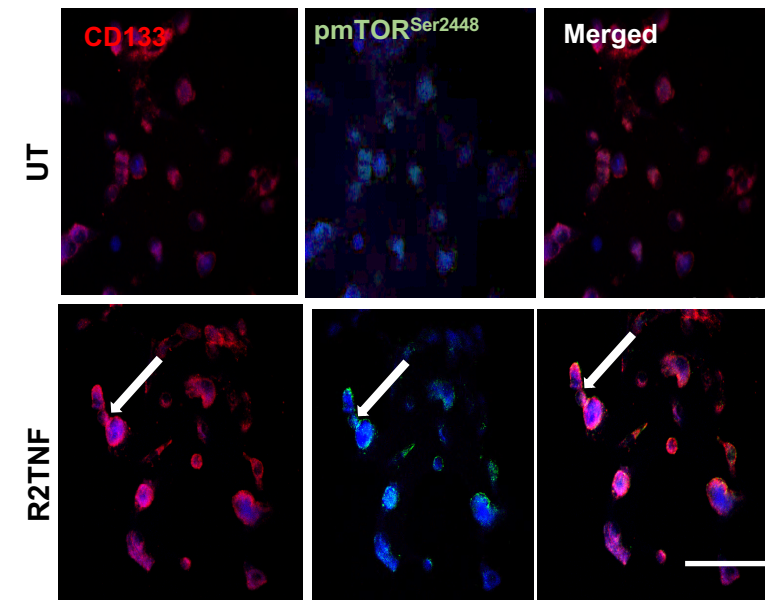

**B**

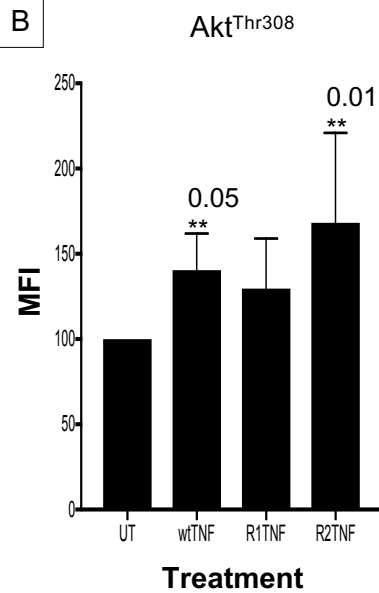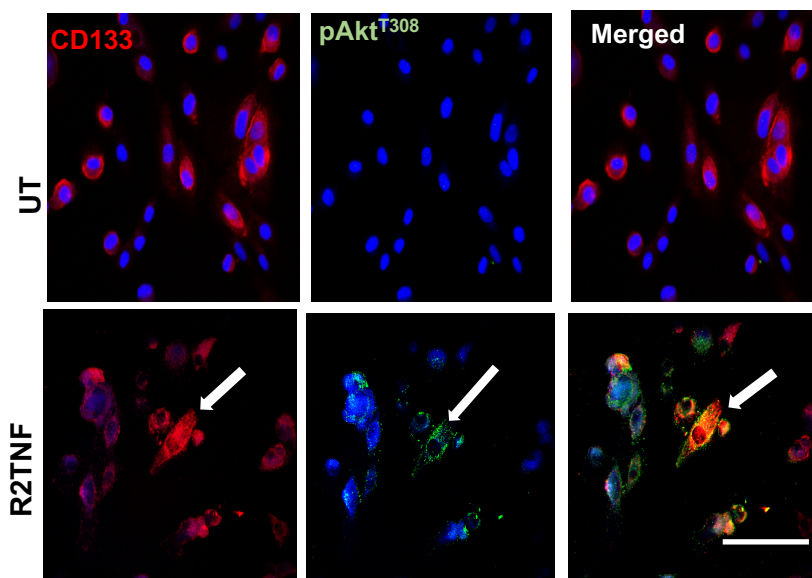

**D**

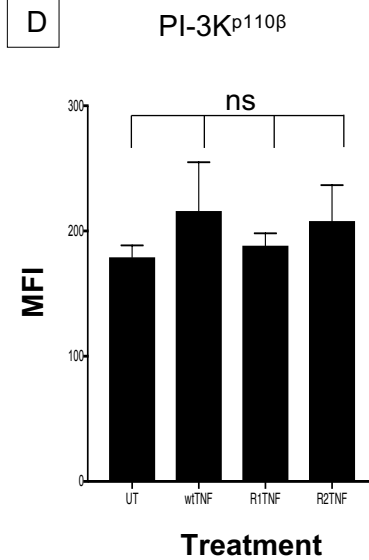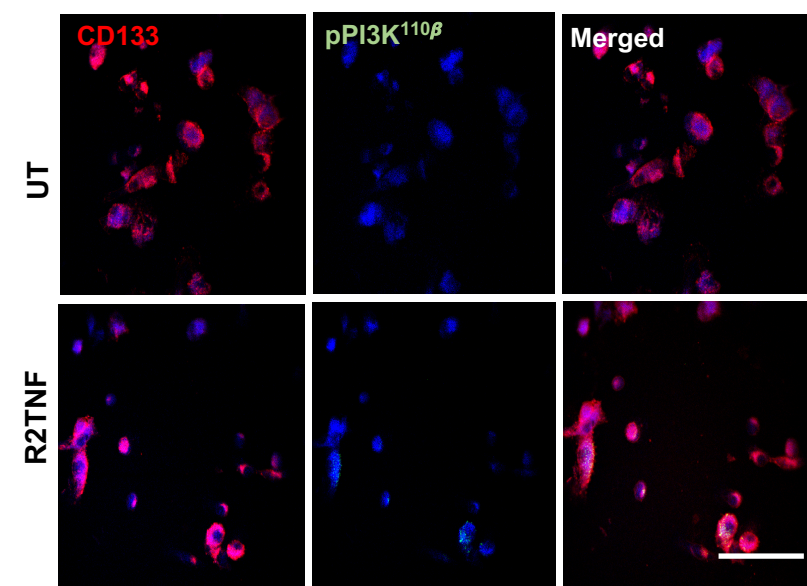

Supplement: Supplementary file 5 [file FBA2-2-126-s005.pdf]

Supplementary Figure 6.

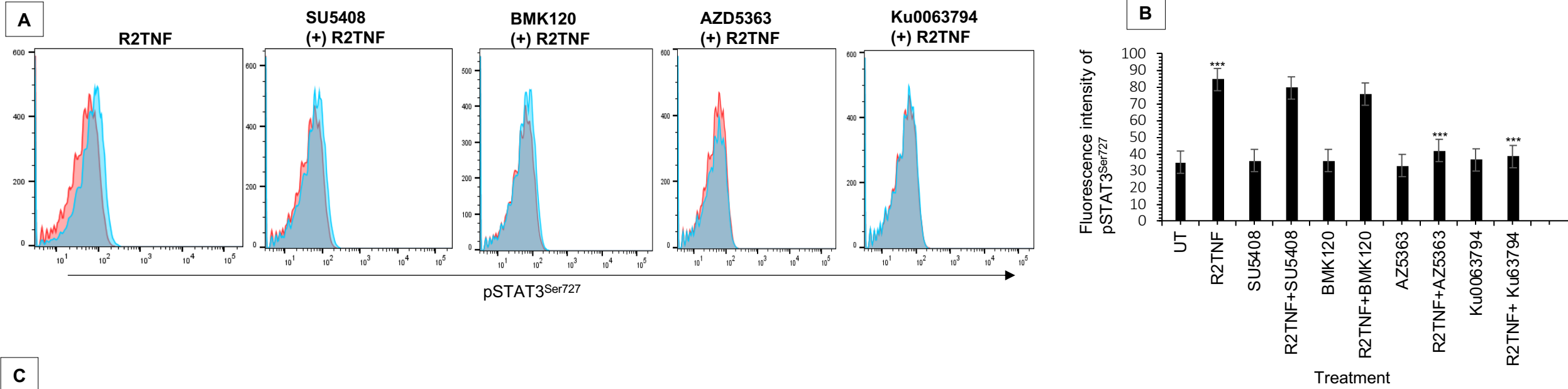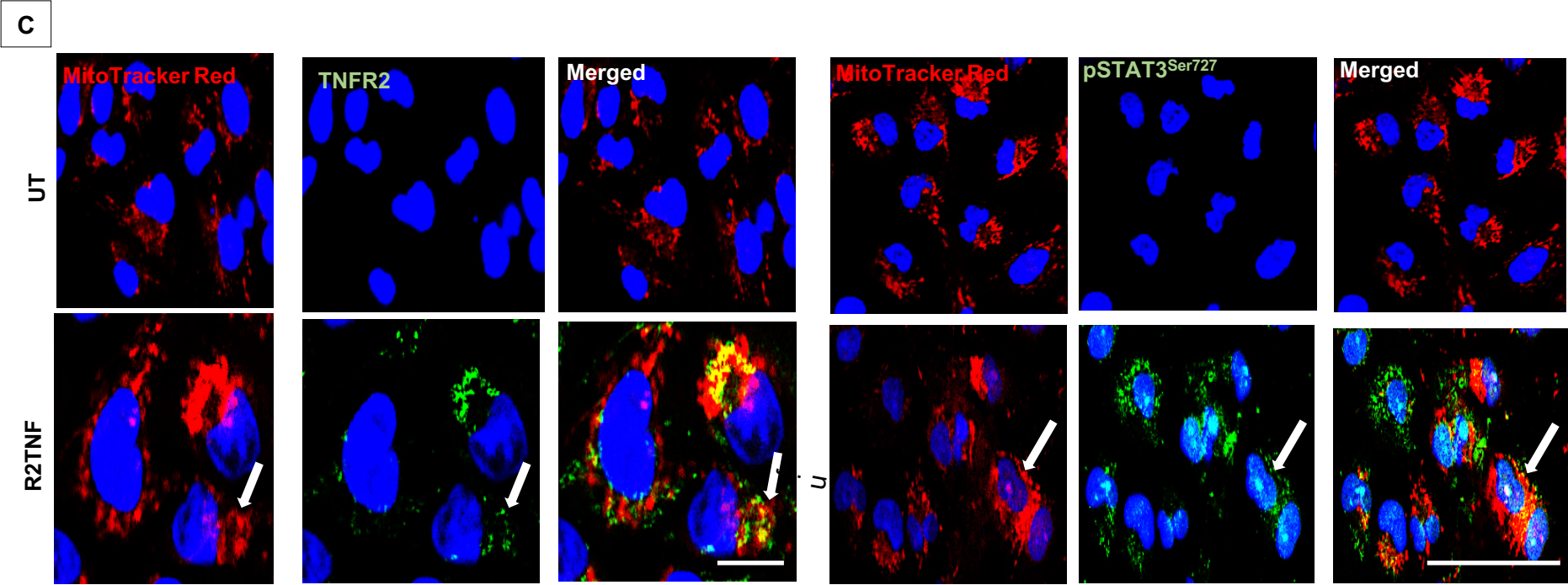

Supplement: Supplementary file 6 [file FBA2-2-126-s006.pdf]

Supplementary Figure 7.

A

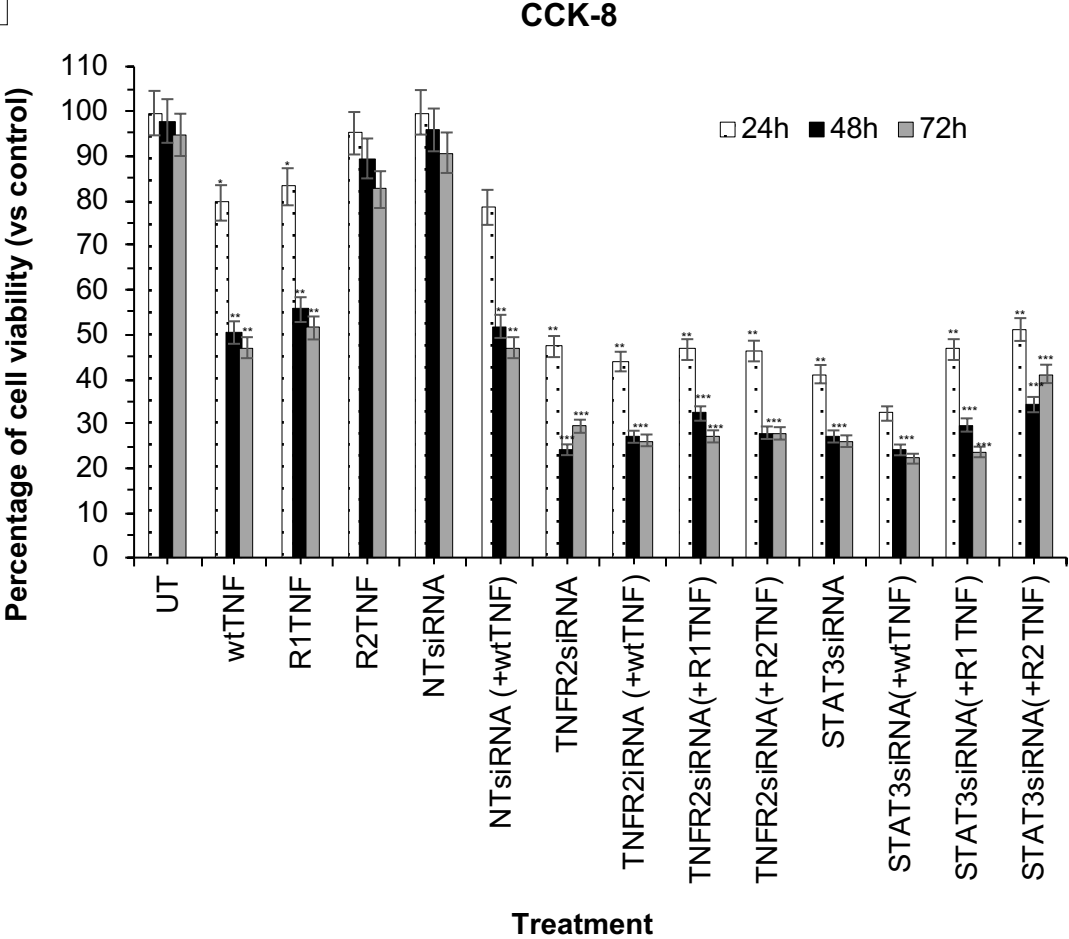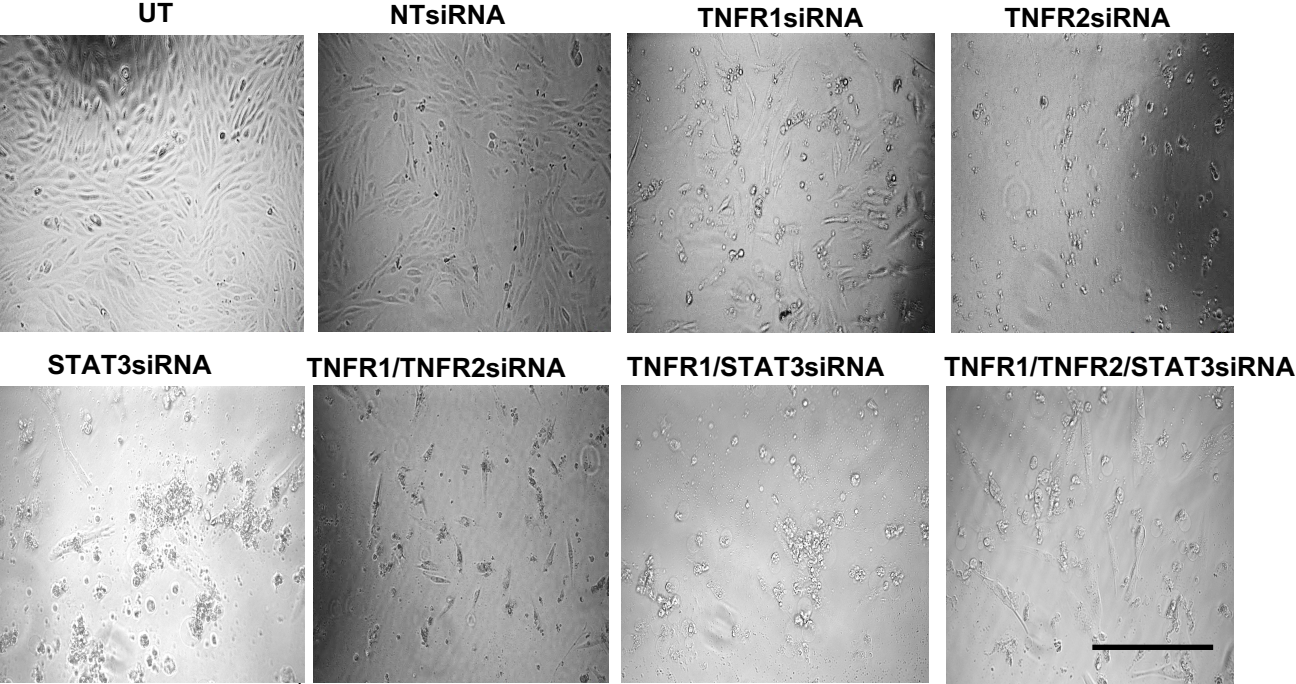

C

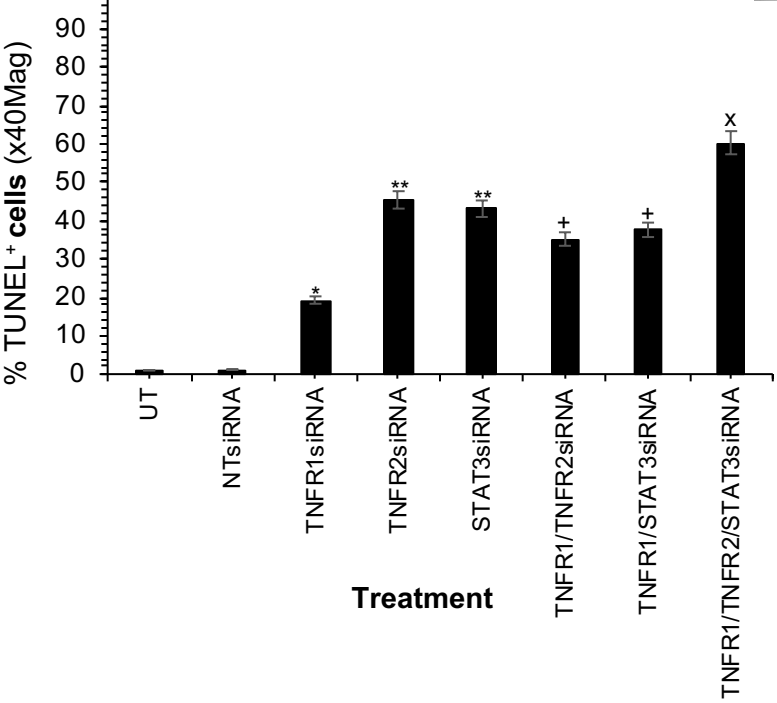

Supplement: Supplementary file 7 [file FBA2-2-126-s007.pdf]

Supplementary Figure 8.

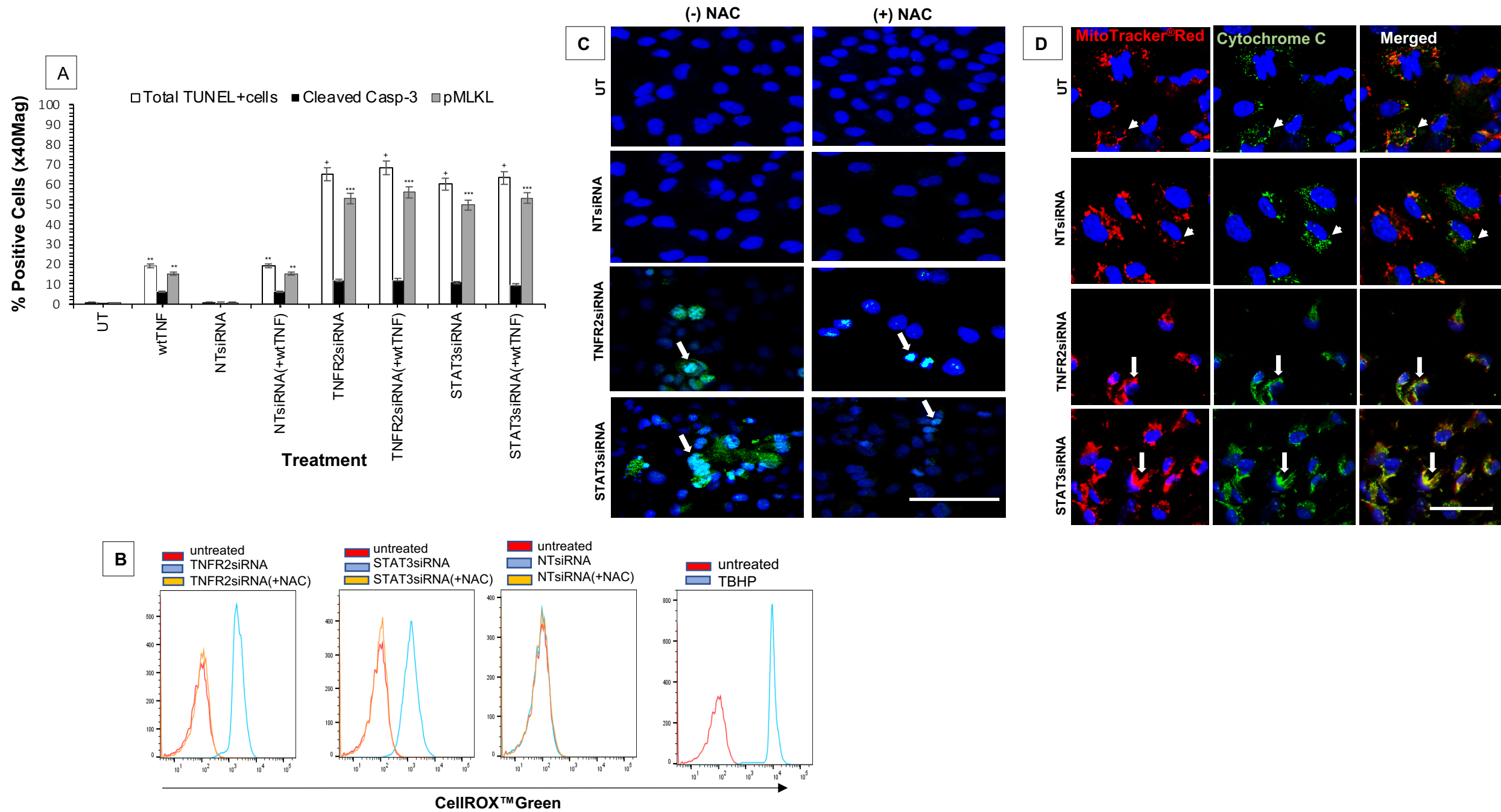

Supplement: Supplementary file 8 [file FBA2-2-126-s008.pdf]
