# Supplementary material for: Tumor necrosis factor receptor‐2 signaling pathways promote survival of cancer stem‐like CD133+ cells in clear cell renal carcinoma
Source: FASEB Bioadv. 2020 Jan 3;2(2):126–44. doi: 10.1096/fba.2019-00071 (PMC7003657; doi:10.1096/fba.2019-00071)
Supplement: Supplementary file 9 [file FBA2-2-126-s009.docx]

**Supplementary Table 1.** Quantification of percentage of cell death (detected by TUNEL) in isolates of NK-CD133^+^cells calculated as positive cells//total number of cells (x100) x40 Mag. Cells were treated with either wild-type(wt)TNF, R1TNF or R2TNF alone or treated in vehicle (DMSO, referred to untreated-UT) for 30min or with specific kinase inhibitors alone for 1h or with inhibitors than TNF (37^o^C). Error bars represent mean + SEM. ^**^P<0.001; ^***^P<0.0001 vs UT. N=3 independent experiments of 3 different isolates with similar results.

| TREATMENT | Untreated | wtTNF | R1TNF | R2TNF |
| --- | --- | --- | --- | --- |
| UT | 2.2+1.2% | 25.8+1.01%^**^ | 27.1+0.7%^**^ | 3.07+0.1% |
| SU5408 | 37.3+0.1%^***^ | 41.3+0.1%^***^ | 45.4+0.3%^***^ | 39.2+0.6% |
| BMK120 | 35.6+0.6%^***^ | 38.6+0.6%^***^ | 42.3+0.4%^***^ | 37.1+0.8% |
| AZ5363 | 35.2+0.9%^***^ | 39.2+0.9%^***^ | 41.0+0.3%^***^ | 36.2+0.1% |
| Ku00063794 | 35.0+0.8%^***^ | 37.0+0.8%^***^ | 43.3+0.1%^***^ | 39.7+0.6% |

DMSO (Dimethyl Sulfoxide) SU5408 (VEGFR2,1𝛍M), BMK120 (Buparlisib; PI3K inhibitor, 4uM), AZD5363(Akt inhibitor, 0.8𝛍M), Ku0063794 (mTORC1/2, 5𝛍M). UT-Untreated, wild-type TNF (wtTNF, 10ng/lml), R1TNF and R2TNF (wild type TNF muteins that bind selectively to TNFR1 or TNFR2 (1𝛍g/ml).
