# Supplementary material for: Tumor necrosis factor receptor‐2 signaling pathways promote survival of cancer stem‐like CD133+ cells in clear cell renal carcinoma
Source: FASEB Bioadv. 2020 Jan 3;2(2):126–44. doi: 10.1096/fba.2019-00071 (PMC7003657; doi:10.1096/fba.2019-00071)
Supplement: Supplementary file 10 [file FBA2-2-126-s010.docx]

**Supplementary Figure Legends.**

**Supplementary Figure 1.** (A). Representative immunoblot confirms knockdown expression of STAT3 and TNFR2 in isolates of ccRCC-^CD133+^CSCs following siRNA transfected targeting STAT3 or TNFR2 (^+^wild-type (wt)TNF) or wtTNF alone at 72h (37^o^C) as compared to control groups (nontargeting siRNA-NTsiRNA and untreated-UT, left in media alone). (B). CCK-8 assay quantified as percentage of cell viability, shows reduced viability in cells treated with R1TNF or wtTNF as compared to R2TNF or control groups. Cell viability is further reduced by the absence of STAT3 or TNFR2, more pronounced at 72h (^+/-^wtTNF, R1TNF or R2TNF); ^**^P<0.01, ^***^P<0.001, ^+^P<0.0001 vs control group. (C). Representative immunoblot confirms knockdown expression of TNFR1 in isolated of ccRCC-^CD133+^CSCs transfected with siRNA targeting TNFR1 for 24h at 37^o^C as compared to cells treated with wtTNF alone. (D). Representative phase-contrast micrographs demonstrate cell death induced by knockdown expression of all the 3 proteins. One way ANOVA. Error bars represent mean + S.E.M; N=3 independent experiments of 3 different isolates with similar results. Scale bars=500µM.

**Supplementary Figure 2.** Representative confocal images of TUNEL combined with immunostaining for (A) cleaved caspase3^P175^ or (B) phosphorylated serine 358 mixed lineage-like domain like pseudo kinase (pMLKL^Ser358^) in isolates of ccRCC-^CD133+^CSCs treated with or without z-VAD.fmk (pan-caspase inhibitor) or Necrostatin-1 (Nec-1, blocks RIPK3, upstream of MLKL) prior to siRNA transfection targeting TNFR2 or STAT3 or control groups (nontargeting siRNA-NTsiRNA or untreated-UT, left in media alone) for 72h at 37^o^C. Knockdown expression of TNFR2 and of STAT3 signals induced cell death as compared to control groups indicated by green-labeled cells with some cells also positive for cleaved caspase-3^p175^ or pMLKL^Ser358^ (*arrows)*. wtTNF alone also induced cell death but at a smaller scale as compared to that induced by the absence of TNFR2 or STAT3 signals.

z-VAD.fmk conferred partial protection, further attenuated by Nec-1. Blue nuclei stained with Hoechst 33342. N=3 independent experiments of 3 different isolates with similar results. Mag x63, Scale bars=100µM.

**Supplementary Figure 3.** (A-D). Organ cultures of normal human kidney were treated with wild type-(wt)TNF, R1TNF, R2TNF or left untreated (UT-in media alone) for 3h at 37^o^C then immunostained for phosphorylation STAT3 serine (pSTAT3^Ser727^) or tyrosine (pSTAT3^Ty705^) and CD133 or with TNFR2 and pSTAT3^Ser727^. (A). Immunofluorescence data quantified as median fluorescence intensity (MFI) shows wtTNF and R2TNF but not R1TNF induction of pSTAT3^Ser727^ as compared to UT control and, immunoreactive cells (*arrows*) demonstrated by confocal microscopy (B). wtTNF and R2TNF also induced co-localization of TNFR2 and pSTAT3^Ser727^ (C), quantified as percentage of positive cells/total number of cells (x100) at x40Mag (D). Error bars represent mean ± SEM; N=5 independent experiments of different organ cultures per treatment group. ^**^P<0.001 *vs* UT; One way ANOVA.

(E). Isolates of NK-^CD133+^ cells show expression of CD133, illustrated by flowcytometry (E), and are positive for stem cell markers (SSAE-4 or Nanog) shown on representative confocal microscopy (F). Blue nuclei stained with Hoechst 33342. N=3 independent experiments of 3 different isolates with similar results. Mag x63, Scale bars: 100µM.

**Supplementary Figure 4.** Isolates of NK-^CD133+^cells were treated with either wild type-(wt)TNF, R1TNF or R2TNF or left untreated (UT, in media alone) then immunostained for phosphorylated STAT3 serine (pSTAT3^Ser727^) or tyrosine (pSTAT3^Ty705^) alone or co-stained with CD133 (A,B) or CD133 and TNFR2. Flowcytometry data quantified as median fluorescence intensity (MFI) shows wtTNF and R2TNF (not R1TNF) induction of pSTAT3^Ser727^ but not pSTAT3^Ty705^ (A B) and, co-localization of CD133 and pSTAT3^Ser727^ is illustrated in representative confocal images (*arrows*) (C). Representative immunoblot of whole cell lysates, relative to total STAT3 in UT cultures shows wtTNF and R2TNF induction of pSTAT3^Ser727^ and R2TNF induction of TNFR2 and pSTAT3^Ser727^ colocalization is shown in representative confocal images (E), quantified as a percentage of positive cells/total number of cells x100 at 40Mag (F). Blue nuclei stained with Hoechst 33342. One way ANOVA. Error bars represent mean ± SEM; N=3 independent experiments of different isolates per treatment group. ^*^P<0.05 and ^**^P<0.01 *vs* UT; ns-not significant. Mag x63, Scale bars=100µM.

**Supplementary Figure 5.** Isolates of NK-^CD133+^cells were treated with either wild type-(wt)TNF, R1TNF or R2TNF or left untreated (UT, in media alone) then immunostained for phosphorylated VEGFR2^Y1057^, PI-3K^p110β^, Akt^Thr308^ and mTORC^Seri2448^. Flowcytometry data quantified as median fluorescence intensity (MFI) shows wtTNF and R2TNF (not R1TNF) induction of phosphorylated Akt^Thr308^ and mTOR^Ser2448^ (B,C) not of VEGFR2^Y1059^ or PI-3K^p110β^ (A, D) with immunoreactive cells (*arrows*) shown on representative confocal images. Blue nuclei stained with Hoechst 33342. Results are mean ± S.E.M; N=3 independent experiments of 3 different isolates with similar results. ns-not significant. One way ANOVA. Mag x63, Scale bars=100µM.

**Supplementary Figure 6**. Isolates of NK-^CD133+^cells were treated with either R2TNF or left untreated (UT, in DMSO) for 30min or pretreatment for 1h at 37^o^C with specific inhibitors to VEGFR2 (SU5408), PI-3K (BMK120), Akt (AZ5363) and mTORC1/2 (Ku0063794) prior to R2TNF and immunostained for phosphorylated serine STAT3 (pSTAT3^Ser727^). Flowcytometry analysis (A) shows R2TNF induction of pSTAT3^Ser727^ (blue peaks), significantly suppressed by inhibition of Akt (AZ5363) and mTORC1/2 (Ku0063794) but not SU5408 or BMK120, quantified in B. Results represent mean ± S.E.M; ^***^P<0.0001 - vs UT or R2TNF, One way ANOVA. (C). R2TNF-treated cells induce TNFR2 and pSTAT3^Ser727^ expression in mitochondria (*arrows*), labeled with MitotrackerRED. Blue nuclei stained with Hoechst 33342. N=3 independent experiments of 3 different isolates with similar results. Mag x63, Scale bars=100µM.

**Supplementary Figure 7**. (A). Quantification of CCK-8 assay on isolates of NK^-CD133+^cells transfected with siRNA targeting TNFR2, STAT3 or negative controls (non-targeting-NTsiRNA or left untreated- UT, left in media alone) for 24, 48 and 72h (37^o^C) with or without wild-type (wt)TNF, R1TNF or R2TNF or treated with wtTNF, R1TNF or R2TNF alone for 30min. wtTNF and R1TNF treatment caused a significant reduction in cell viability in a time-dependent manner, further attenuated by the absence of TNFR2/STAT3 signals as compared to control groups (NTsiRNA or UT). ^*^P<0.05, ^**^P<0.01, ^***^P<0.001.

(B). Representative phase-contrast micrographs show the extent of cell death induced by the absence of TNFR1 signals as compared to the absence of TNFR2/STAT3 signals versus controls. (C). TUNEL-^positive^ cells quantified as a percentage of cell death induced by the absence of TNFR1, TNFR2, or STAT3. Note, the absence of TNFR2 and/or STAT3 signals induce a high degree of cell death as compared to the absence of TNFR1 signals alone, which is much more pronounced in the absence of all 3 proteins. One way ANOVA. Error bars mean + S.E.M. N=3 independent experiments of 3 different isolates with similar results. ^*^P<0.05 *vs* control group, ^**^P<0.001 *vs* TNFR1siRNA, ^+^P<0.05 *vs* TNFR2siRNA or STAT3siRNA, ^X^P<0.0001 *vs* all siRNA against all 3 molecules. Scale bars=100µM.

**Supplementary Figure 8**. Isolates of NK^-CD133+^cells were transfected with RNA targeting TNFR2 or STAT3 or negative controls (non-targeting-NTsiRNA or left untreated- UT, left in media alone) for 72h (37^o^C) with or without wild-type (wt)TNF or treated with wtTNF alone for 30min at 37^o^C. (A). Cell death quantified as a percentage of TUNEL^-positive^ cells/total cells( x100) at x40Mag shows a high level of cell death in the absence of TNFR2 or STAT3 signals as compared to control groups, which is increased by a small margin by addition of wtTNF. Some of the TUNEL^-positive^ cells are also positive for cleaved caspase-3^p175^ but majority are positive for pMLKL^Ser358^. One way ANOVA. Error bars represent mean + S.E.M; ^**^P<0.01 and ^+^P<0.0001 (white columns) *vs* UT; ^*^P<0.05 (cleaved caspase-3^+^cells within each group), ^**^P<0.01 and ^***^P<0.001 (pMLKL^Ser358+^cells within each groups). (B). Flowcytometry data shows induction of CellROX™Green expression (Blue) in the absence of TNFR2 or STAT3 signals as compared to negative control groups, diminished by anti-oxidant N-Acetyl Cysteine (NAC) (Orange). UT controls (Red), positive controls (Blue-treated with ROS inducer Tert-butly hydroperoxide-TBHP). (C). Representative confocal images show nuclear expression of CellROX™Green, diminished by NAC (*arrows*).

(D). The absence of TNFR2 or STAT3 signals also result in cytochrome c release in cytosol *(small arrows)* which show a diffuse staining pattern as compared to a define and punctate pattern in control groups (*arrowheads*). Blue nuclei stained with Hoechst 33342. N=3 independent experiments of 3 different isolates with similar results. Scale bars, C=100µM, D=75µM.
